# Supplementary material for: A cocktail nanovaccine targeting key entry glycoproteins elicits high neutralizing antibody levels against EBV infection
Source: Nat Commun. 2024 Jun 21;15:5310. doi: 10.1038/s41467-024-49546-w (PMC11192767; doi:10.1038/s41467-024-49546-w)
Supplement: Supplementary file 1 — supplementary information [file 41467_2024_49546_MOESM1_ESM.pdf]

A cocktail nanovaccine targeting key entry glycoproteins elicits high neutralizing antibody levels against EBV infection

Supplementary Information

Supplementary figures and figure legends

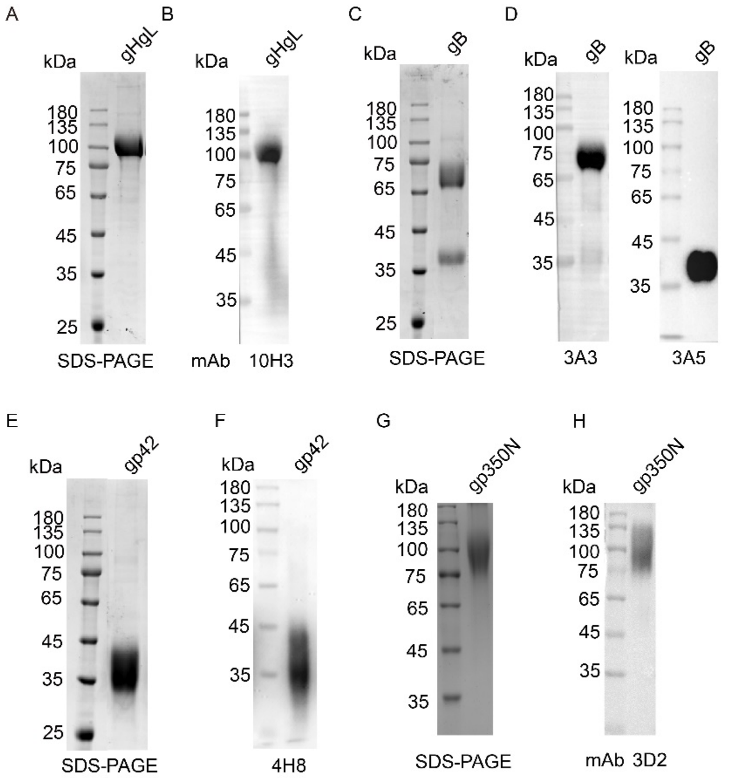

Figure S1. Expression and purification of EBV gHgL, gB, gp42 and gp350.

(A) SDS-PAGE analysis of purified proteins gHgL expressed in 293F cells. Gels were stained with Coomassie brilliant blue (n=3).

(B) Western blot analysis of gHgL detected by mAb 10H3 under reducing SDS-PAGE (n=3).

(C) SDS-PAGE analysis of purified proteins gB expressed in 293F cells. Gels were stained with Coomassie brilliant blue (n=3).

(D) Western blot analysis of gB detected by mAbs 3A3 and 3A5 under reducing SDS-PAGE. mAb 3A3 bound the ~ 70 kDa fragment and 3A5 bound the ~ 40 kDa fragment (n=3).

(E) SDS-PAGE analysis of purified proteins gp42 expressed in 293F cells. Gels were stained with Coomassie brilliant blue (n=3).

(F) Western blot analysis of gp42 detected by mAb 4H8 [1] under reducing SDS-PAGE (n=3).

(G) SDS-PAGE analysis of purified proteins gp350N expressed in 293F cells. Gels were stained with Coomassie brilliant blue (n=3).

(H) Western blot analysis of gp350N detected by mAb 3D2 under reducing SDS-PAGE (n=3).

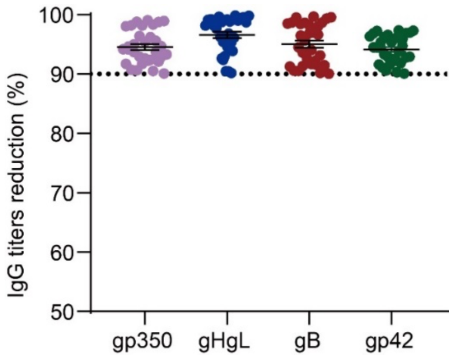

Figure S2. Reduction of glycoprotein-specific IgG titers after depletion by cells overexpressing specific glycoprotein in sera from 32 healthy EBV carriers. The percentage of IgG titer reduction was calculated by the equation (1-IgG titer-depleted/IgG titer-before) × 100%. Source data are provided as a Source Data file.

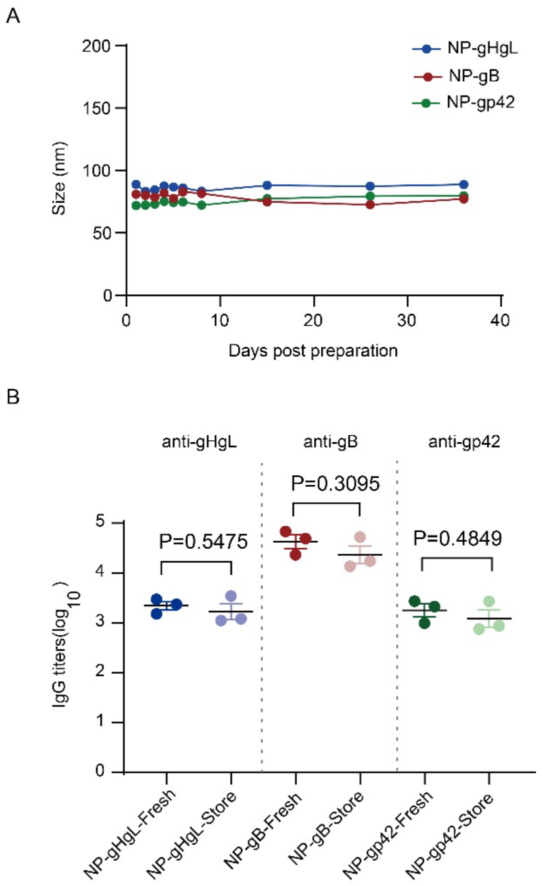

Figure S3. Size and immunogenicity stability of nanovaccines.

(A) Size of nanoparticle vaccines stored at 4°C. Data are shown as the mean of three independent replicates.

(B) Total IgG titers of sera collected from C57BL/6J mice immunized once with nanovaccines that were freshly prepared or stored at 4°C for 30 days were not significantly different. Data are shown as mean ± SEM (n=3). P values calculated using unpaired two-tailed Welch's t test are shown as precise values. Source data are provided as a Source Data file.

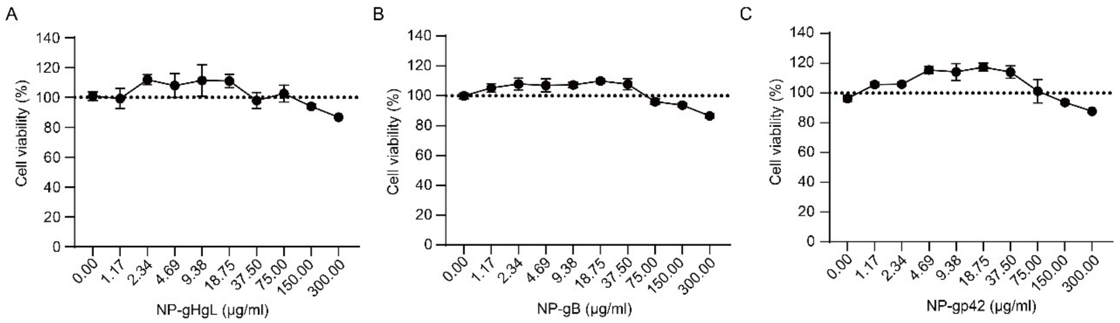

Figure S4. Viability of DC2.4 cells treated with different concentrations of NP-gHgL (A), NP-gB (B) and NP-gp42 (C) for 24 h. Data are shown as mean ± SEM (n=4). Source data are provided as a Source Data file.

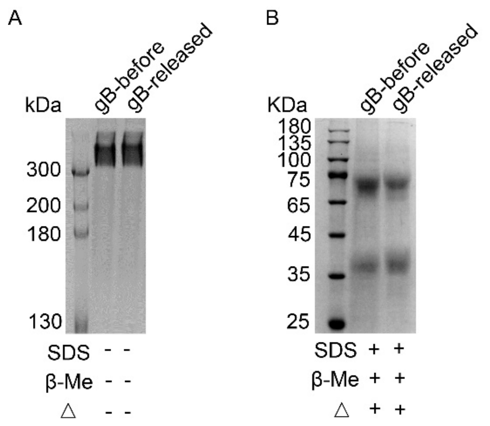

Figure S5. Non-reducing native PAGE (A) and reducing SDS-PAGE (B) analysis of gB before encapsulation and gB released from nanoparticles (n=3).
Δ denotes heating at 100°C for 10 min. One band with molecular weight of ~330 kDa was observed in non-reducing native PAGE. Two bands with molecular weights of ~ 70 kDa and ~ 40 kDa were observed after reducing SDS-PAGE.

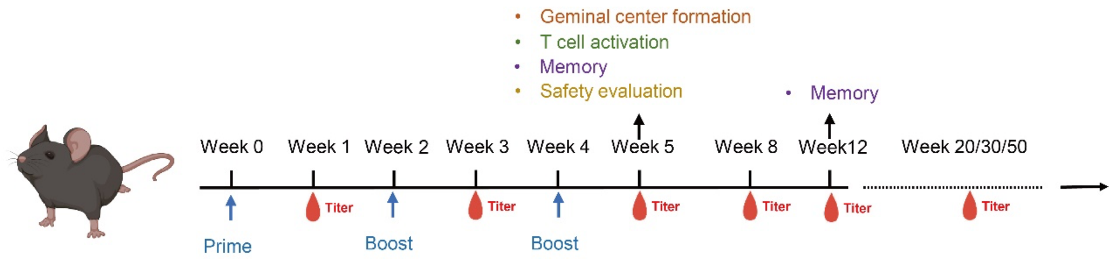

Figure S6. Diagram of C57BL/6J mice immunization schedule. The figure was created from Biorender.com.

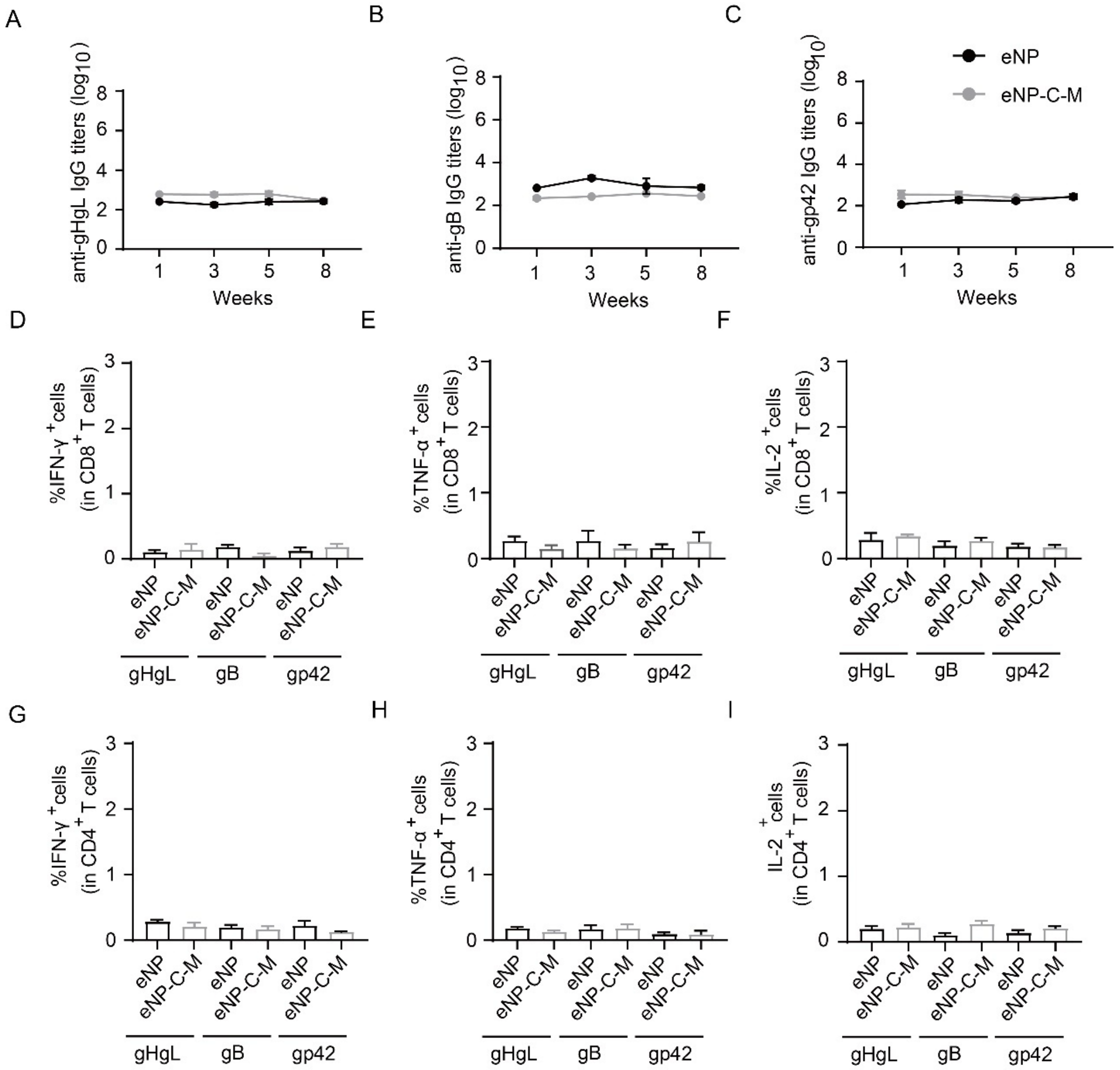

Figure S7. Empty nanoparticles or empty nanoparticles with adjuvants are non-immunogenic in C57BL/6J mice.

(A-C) Anti-gHgL (A), anti-gB (B) and anti-gp42 (C) IgG titers of sera collected from C57BL/6J mice immunized with empty nanoparticles (eNP) or empty nanoparticles containing CpG and MPLA (eNP-C-M) (n=3). Antigen-specific total IgG titers were detected by ELISA (n=3).

(D-F) Percentage of IFN-γ^+ $CD8^+$ T cells (D), TNF-α^+ $CD8^+$ T cells (E) and IL-2^+ $CD8^+$ T cells (F) in splenic lymphocytes restimulated with gHgL, gB or gp42 antigens *in vitro* (n=3).

(G-I) Percentage of IFN-γ^+ $CD4^+$ T cells (G), TNF-α^+ $CD4^+$ T cells (H) and IL-2^+ $CD4^+$ T cells (I) in splenic lymphocytes restimulated with gHgL, gB or gp42 antigens *in vitro* (n=3).

Data are shown as mean ± SEM. Source data are provided as a Source Data file.

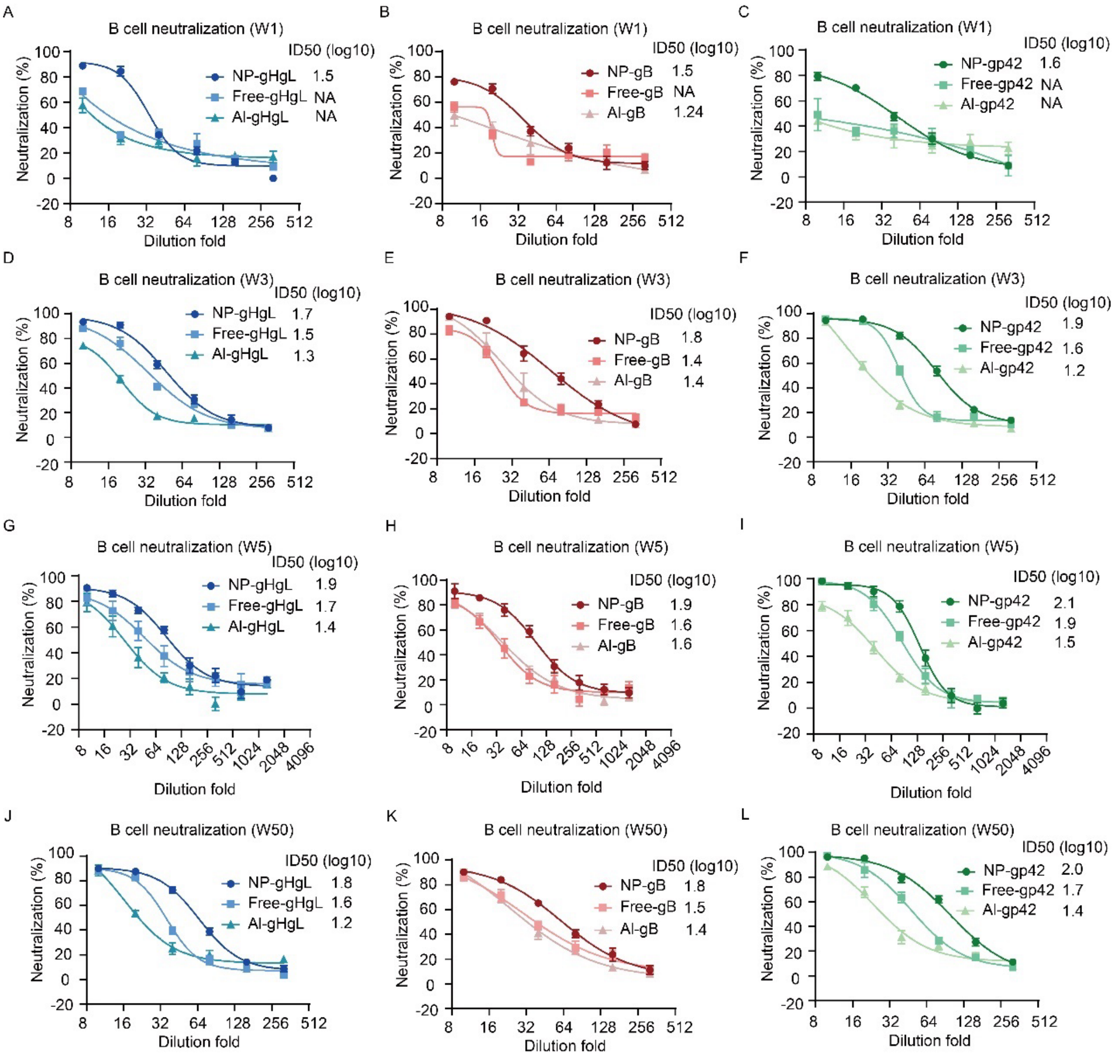

Figure S8. B cell infection neutralizing abilities of sera from mice immunized with various vaccine formulations collected at different time points. Neutralization of CNE2-EBV-GFP infection of Akata B cells was tested.

(A-C) B cell infection neutralizing titers of sera collected at week 1 from mice immunized with gHgL-based vaccines (A), gB-based vaccines (B) and gp42-based vaccines (C), respectively.

(D-F) B cell infection neutralizing titers of sera collected at week 3 from mice immunized with gHgL-based vaccines (D), gB-based vaccines (E) and gp42-based vaccines (F) , respectively.

(G-I) B cell infection neutralizing titers of sera collected at week 5 from mice immunized with gHgL-based vaccines (G), gB-based vaccines (H) and gp42-based vaccines (I), respectively.

(J-L) B cell infection neutralizing titers of sera collected at week 50 from mice immunized with gHgL-based vaccines (J), gB-based vaccines (K) and gp42-based vaccines (L) , respectively.
Half maximal inhibitory dilution fold (ID50) was calculated by sigmoid trend fitting.
Data points are shown as the mean ± SEM (n=5). Source data are provided as a Source Data file.

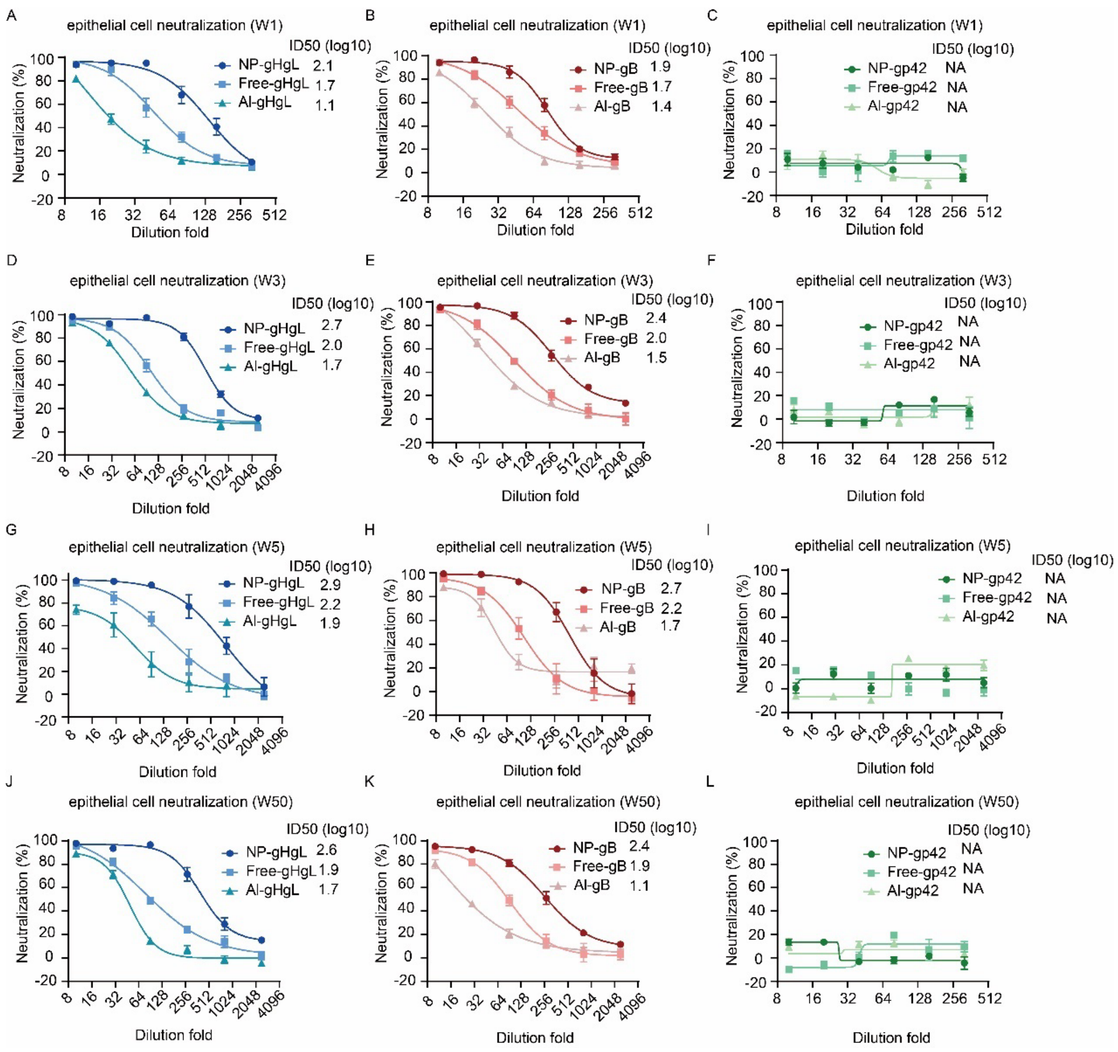

Figure S9. Epithelial cell infection neutralizing abilities of sera from mice immunized with various vaccine formulations collected at different time points. Neutralization Akata-EBV-GFP infection of HNE1 cells was tested.

(A-C) Epithelial cell infection neutralizing titers of sera collected at week 1 from mice immunized with gHgL-based vaccines (A), gB-based vaccines (B) and gp42-based vaccines (C), respectively.

(D-F) Epithelial cell infection neutralizing titers of sera collected at week 3 from mice immunized with gHgL-based vaccines (D), gB-based vaccines (E) and gp42-based vaccines (F), respectively.

(G-I) Epithelial cell infection neutralizing titers of sera collected at week 5 from mice immunized with gHgL-based vaccines (G), gB-based vaccines (H) and gp42-based vaccines (I), respectively.

(J-L) Epithelial cell infection neutralizing titers of sera collected at week 50 from mice immunized with gHgL-based vaccines (J), gB-based vaccines (K) and gp42-based vaccines (L), respectively.

Half maximal inhibitory dilution fold (ID50) was calculated by sigmoid trend fitting. Data points are shown as the mean ± SEM (n=5). Source data are provided as a Source Data file.

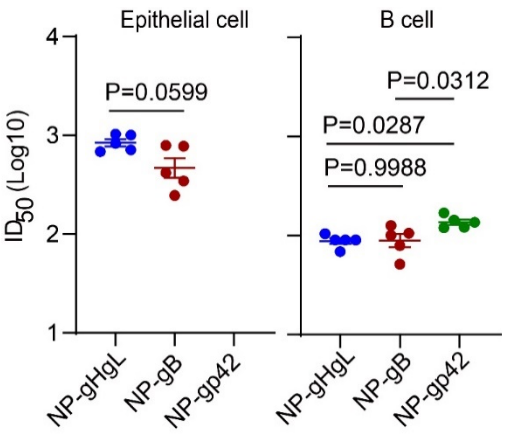

Figure S10. Comparison of epithelial cell (left panel) and B cell (right panel) neutralizing titers of sera elicited by different nanovaccines.

Data are shown as the mean ± SEM (n=5). Left panel: P values calculated using unpaired two-tailed Welch's t test are shown as precise values (n=5). Right panel: P values calculated using one-way ANOVA with Turkey's multiple comparison are shown as precise values..

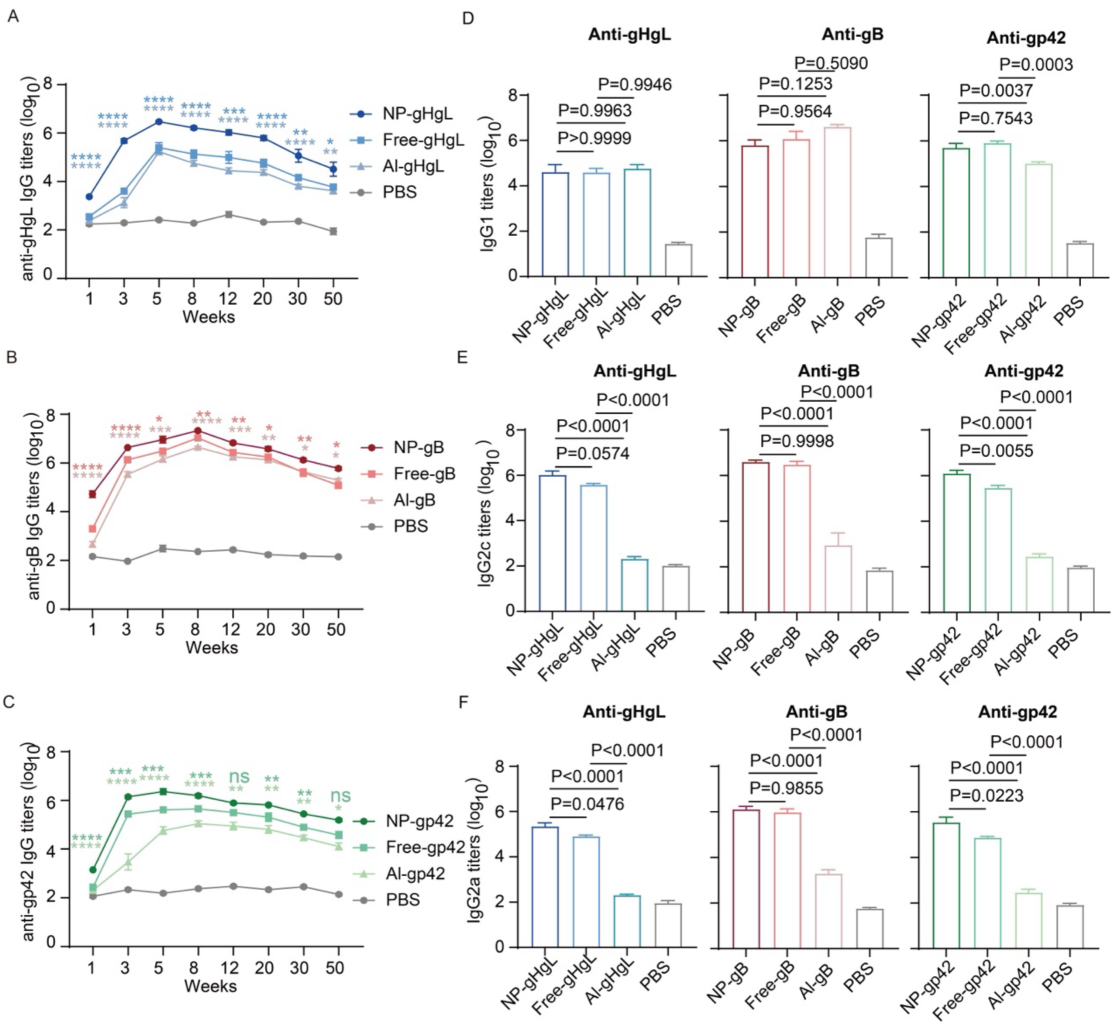

Figure S11. Titers of IgG subtypes induced by different vaccine formulations in C57BL/6J mice.

(A-C) Total IgG titers of sera collected from C57BL/6J mice immunized with different vaccine formulations. (A) Anti-gHgL IgG titers induced by NP-gHgL, Free-gHgL, Al-gHgL and PBS, respectively. (B) Anti-gB IgG titers induced by NP-gB, Free-gB, Al-gB and PBS, respectively. (C) Anti-gp42 IgG titers induced by NP-gp42, Free-gp42, Al-gp42 and PBS, respectively. Data are shown as mean ± SEM (n=5). Statistical analysis was performed using one-way ANOVA with Dunnett's multiple comparison. The color of the asterisks or ns denotes statistical difference with the NP-formation. P values are shown in the source data file.

(D-F) Anti-gHgL (left panel), anti-gB (middle panel) and anti-gp42 (right panel) IgG1 titers (D), IgG2c titers (E) and IgG2a titers (F) of sera from C57BL/6J mice immunized with different vaccine formulations on day 35. Data are shown as mean ±

SEM (n=5). P values calculated using one-way ANOVA with Turkey’s multiple comparison are shown as precise values. Source data are provided as a Source Data file.

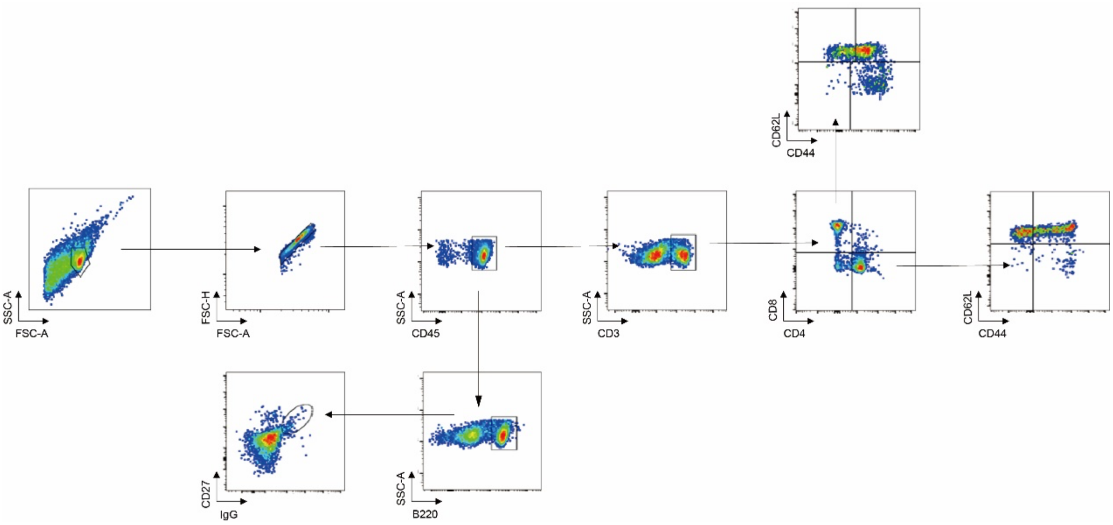

Figure S12. Representative flow cytometry plots showing the gating strategy used to analyze effector memory T cells and memory B cells (correspond to figure 2H, 2L, S13 and S23).

Memory B cells: $CD27^{+}IgG^{+}$; Effector memory T cells: $CD44^{hi}CD62L^{low}$

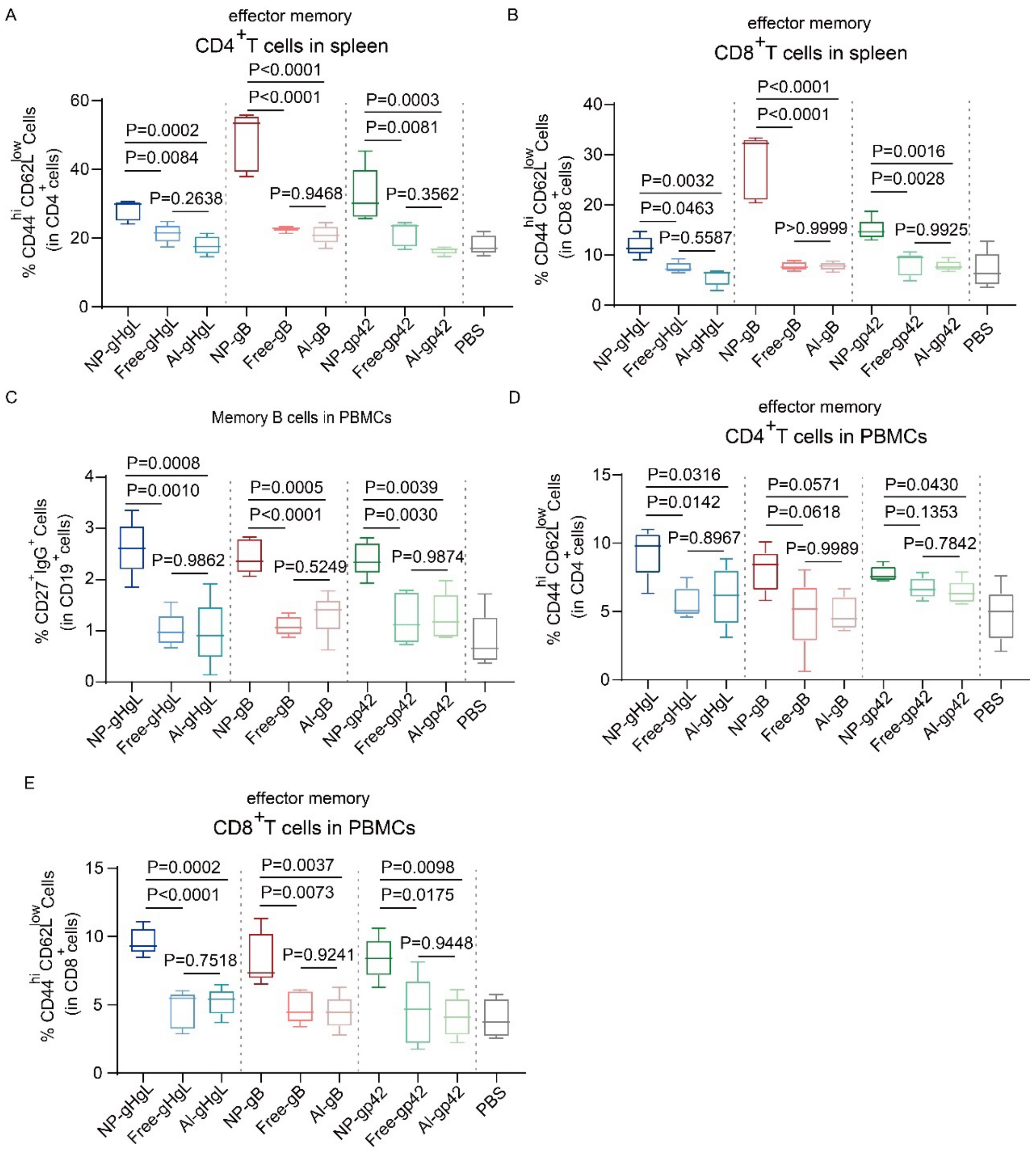

Figure S13. Generation of memory cells generation after immunization.

(A-B) Effector memory (EM) $CD4^+$ T cells (A) and $CD8^+$ T cells (B) in the spleen on day 35 were evaluated by flow cytometry (n=5).

(C-E) Memory B cells (C) and effector memory $CD4^+$ T cells (D) and $CD8^+$ T cells (E) in peripheral blood were detected 8 weeks after the third immunization (n=5). P values calculated using one-way ANOVA with Turkey's multiple comparison are shown as precise values.

Source data are provided as a Source Data file. The center line indicates the median, upper and lower box lines show quartiles; and whiskers show the maximum and minimum values.

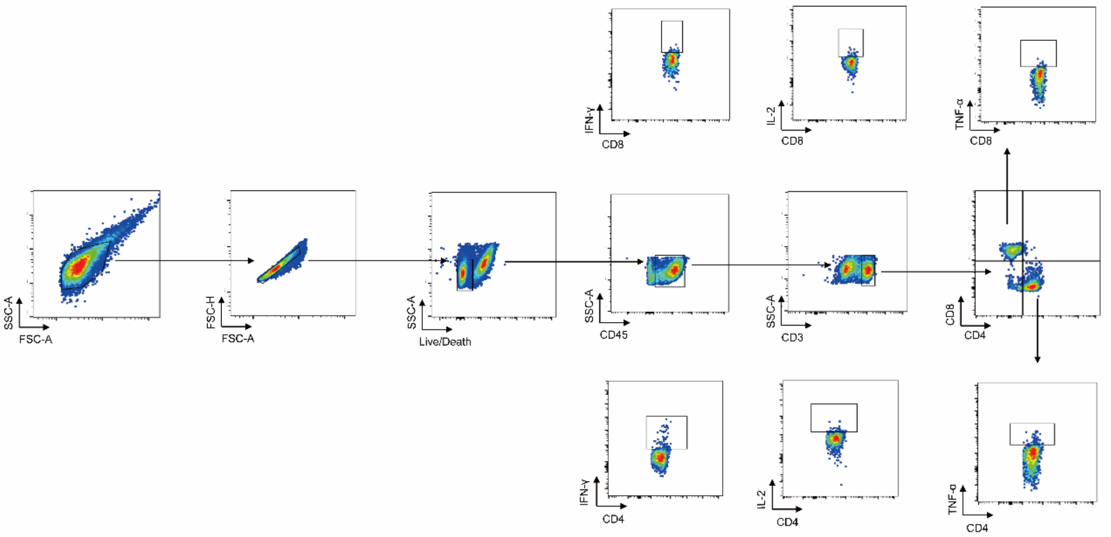

Figure S14. Representative flow cytometry plots showing the gating strategy used to analyze antigen specific T cell immune responses (correspond to Figure 2I-2K, 4D, S7 and S18C).

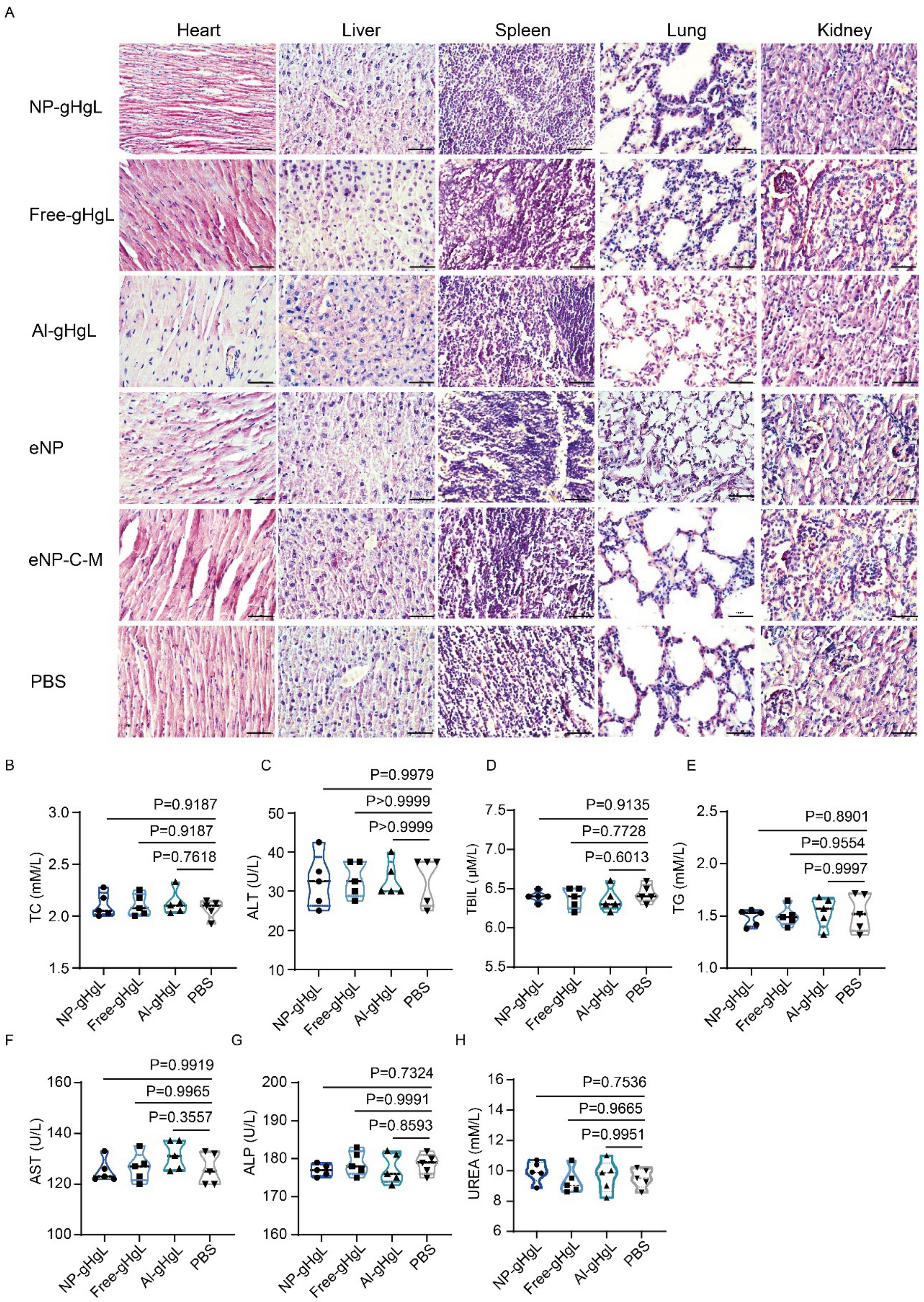

Figure S15. Safety of Nanovaccines in C57BL/6J mice.

(A) Representative C57BL/6J mice heart, liver, spleen, lung and kidney tissues stained with hematoxylin and eosin (HE) at necropsy 1 week after the third immunization with the indicated vaccine formulations (Scale bar=50 μm) (n=5).

(B-G) Levels of TC (B), ALT (C), TBIL (D), TG (E), AST (F), ALP (G) and UREA (H) in the sera from mice immunized with various vaccines. Sera were collected at week 5 (n=5). P values calculated using one-way ANOVA with Dunnett's multiple comparison are shown as precise values. Source data are provided as a Source Data file.

total cholesterol, TC; alanine transaminase, ALT; total bilirubin, TBIL; triglycerides, TG; aspartate aminotransferase, AST; alkaline phosphatase, ALP.

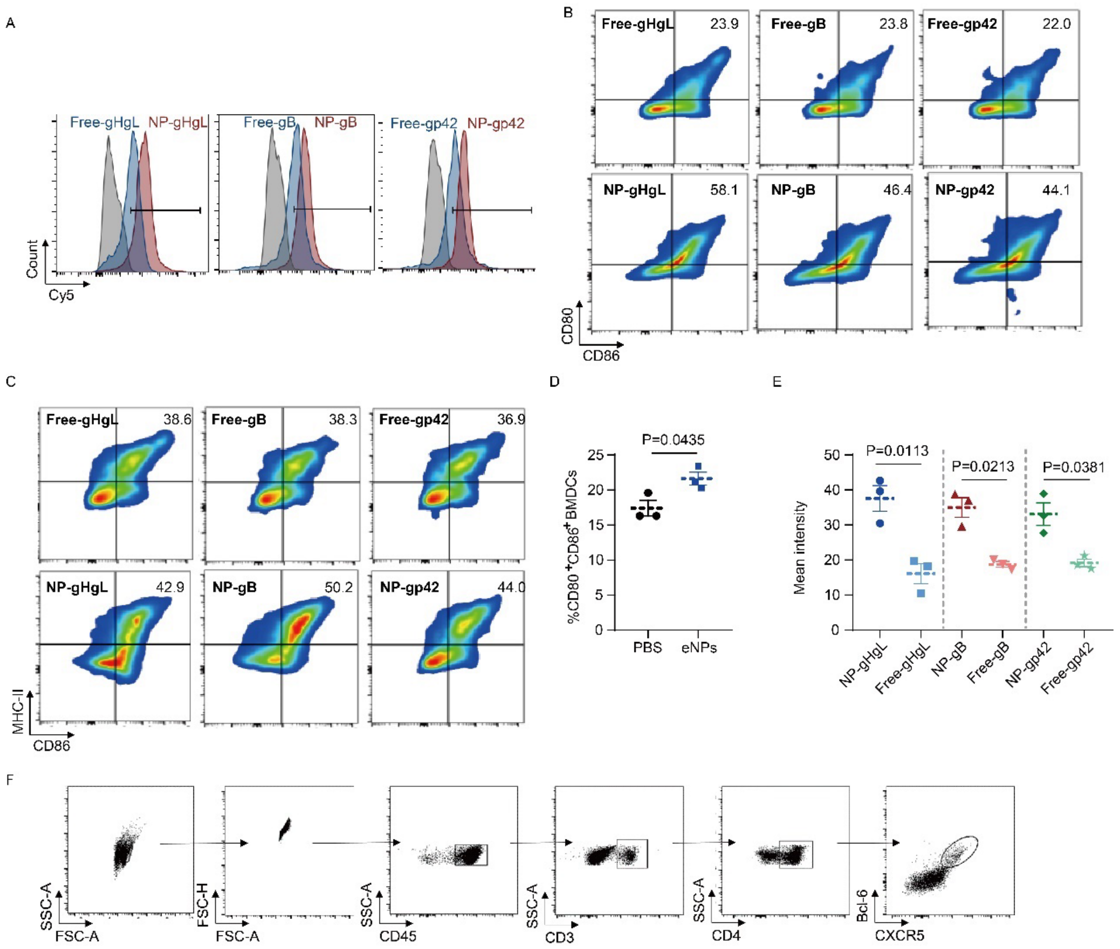

Figure S16. BMDCs internalization and maturation after incubation with different vaccine formulations.

(A) Bone marrow-derived dendritic cells (BMDCs) internalization of free (Free-) or nanoparticle (NP-) formsof Cy5-labeled gHgL, gB and gp42 and two adjuvants was measured by flow cytometry after 4 h incubation at 37°C. PBS was used as negative control (gray histogram).

(B-C) Representative flow cytometry plots of detection of CD80 vs CD86 (B) and MHC-II vs CD86 (C) on BMDCs incubated for 24 h with the indicated vaccine formulations. Numbers indicate the percentage of double-positive cells.

(D) Expression detection (B) of CD80 and CD86 on BMDCs incubated for 24 h with empty nanoparticles (eNPs).

(E) Mean fluorescence intensity of antigens in the draining inguinal lymph nodes 6 h post-immunization. It is the quantification analysis of antigen fluorescence in Figure 3E.

(D-E) Data points are shown as the mean ± SEM (n=3). P values calculated using unpaired two-tailed Welch's t test are shown as precise values. Source data are provided as a Source Data file.

(F) Representative flow cytometry plots showing the gating strategy used to analyze Tfh cells (corresponded to Figure 3F).

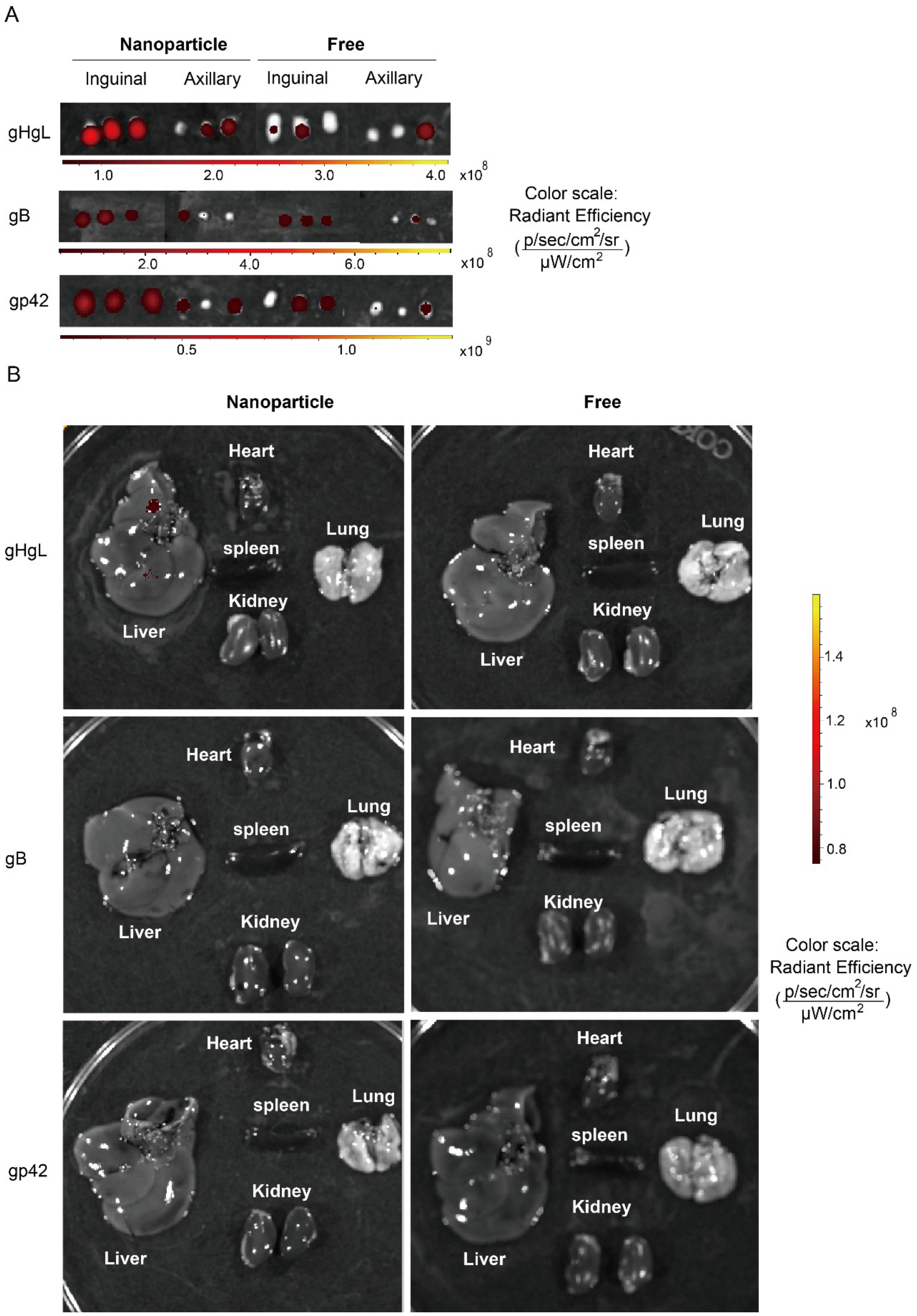

Figure S17. LNs (A) and organs (B) of mice injected subcutaneously with vaccines containing 5 μg Cy5-labeled antigen at 24 h post-injection using the IVIS optical imaging system to detect Cy5 fluorescence (n=3).

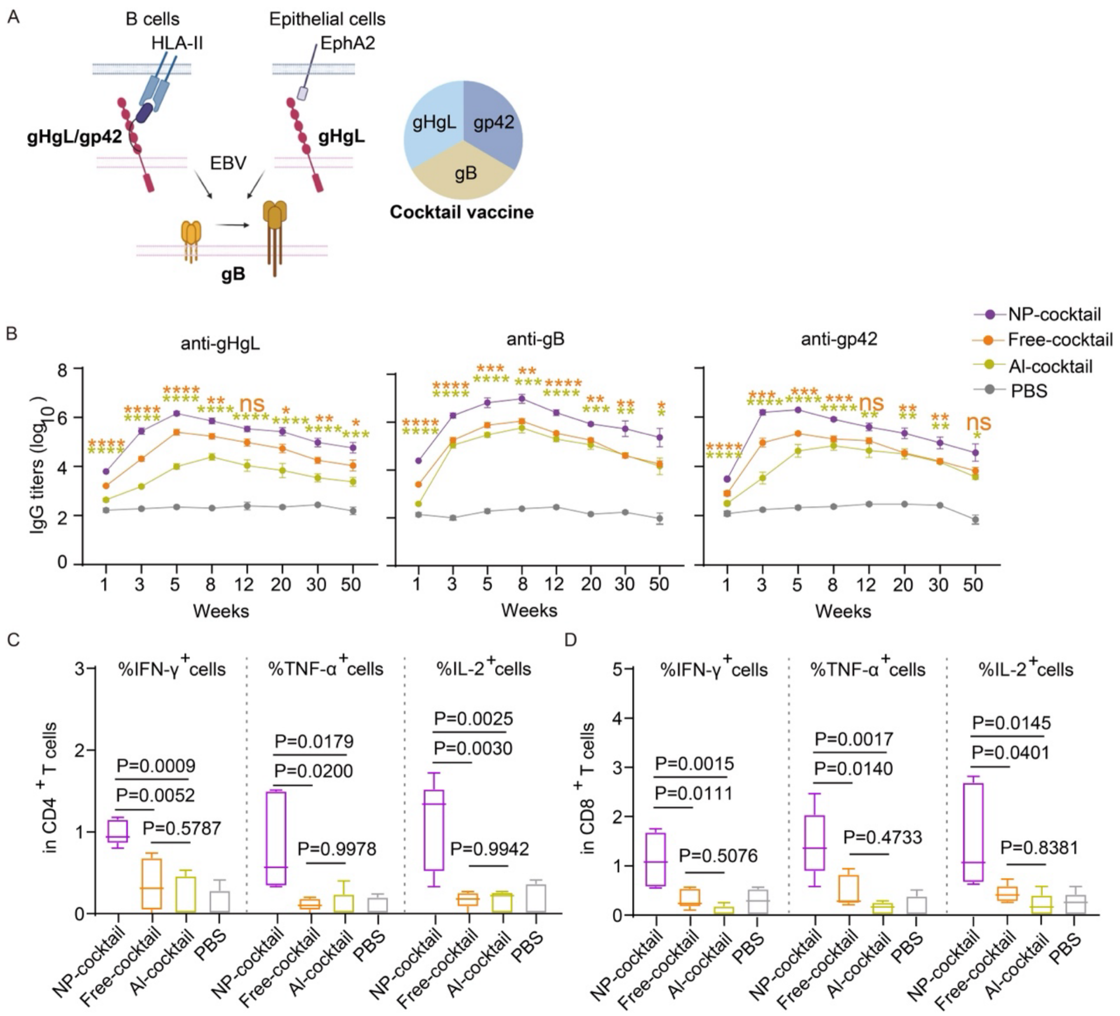

Figure S18. NP-cocktail generated more potent humoral and cellular immune responses.

(A) Schematic diagram of EBV entry processes in B cells and epithelial cells. The pie chart indicates that multi-target cocktail formulations comprise an equal ratio of antigens. The figure was created from Biorender.com.

(B) ELISA data of anti-gHgL IgG titers (left panel), anti-gB IgG titers (middle panel) and anti-gp42 IgG titers (right panel) from sera collected from C57BL/6J mice following immunization with cocktails of the indicated vaccine formulations. Data are shown as mean ± SEM (n=5). Statistical analysis was performed using one-way ANOVA with Dunnett's multiple comparison. The color of the asterisks or ns denotes statistical difference with the NP-formation. P values are shown in the source data file.

(C-D) Antigen-specific $CD4^{+}$ (C) and $CD8^{+}$ (D) T cell responses on day 35 (5 weeks)

in the spleen of mice immunized with the indicated vaccines. Antigen specific T cells were measured by intracellular cytokine staining assay after restimulation with gHgL, gB and gp42 *in vitro* (n=5). P values calculated using one-way ANOVA with Turkey's multiple comparison are shown as precise values. The center line indicates the median, upper and lower box lines show quartiles; and whiskers show the maximum and minimum values.

Source data are provided as a Source Data file.

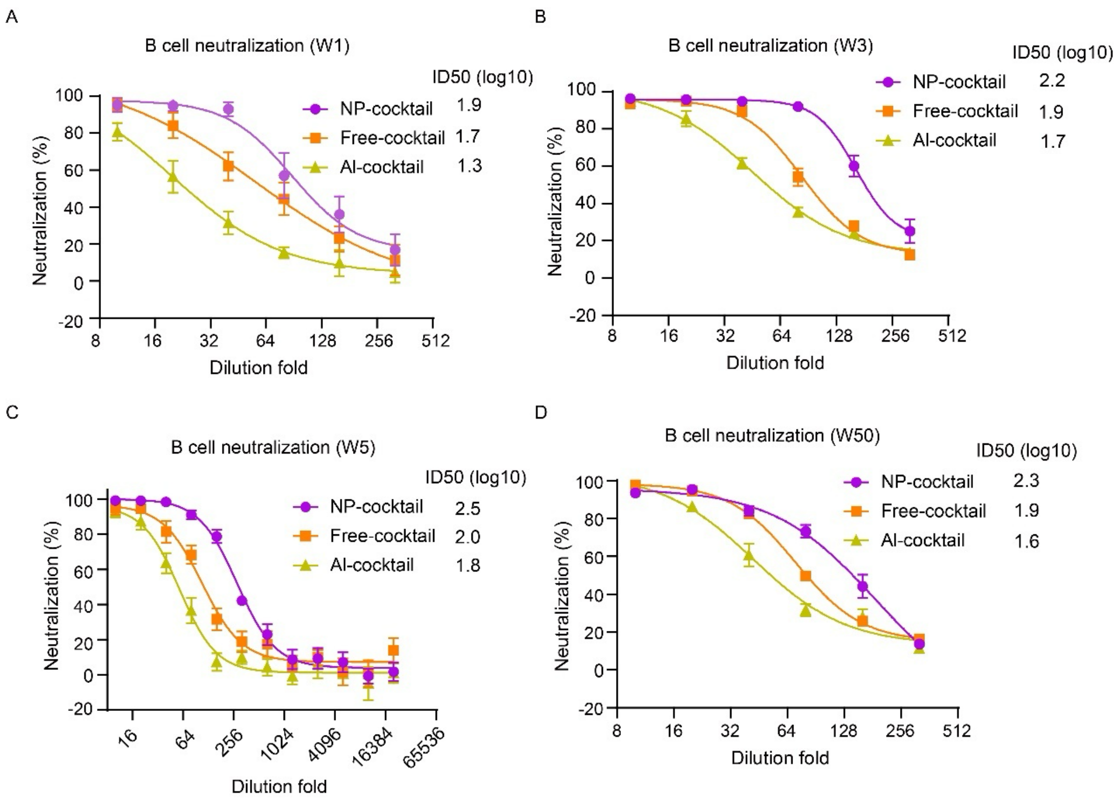

Figure S19. B cell infection neutralizing abilities of sera from mice immunized with cocktail vaccines collected at different time points.

(A) B cell infection neutralizing titers of sera collected at week 1 from mice immunized with NP-cocktail, Free-cocktail and Al-cocktail.

(B) B cell infection neutralizing titers of sera collected at week 3 from mice immunized with NP-cocktail, Free-cocktail and Al-cocktail.

(C) B cell infection neutralizing titers of sera collected at week 5 from mice immunized with NP-cocktail, Free-cocktail and Al-cocktail.

(D) B cell infection neutralizing titers of sera collected at week 50 from mice immunized with NP-cocktail, Free-cocktail and Al-cocktail.

Half maximal inhibitory dilution fold (ID50) was calculated by sigmoid trend fitting. Data points are shown as the mean ± SEM (n=5). Source data are provided as a Source Data file.

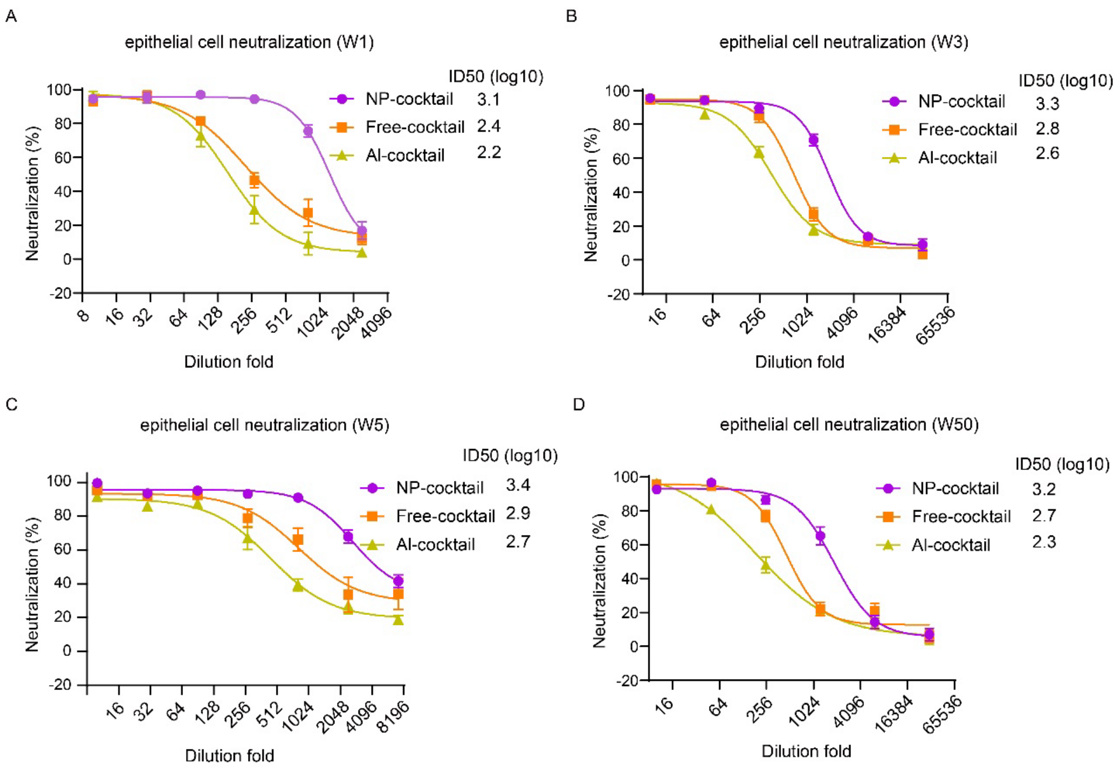

Figure S20. Epithelial cell infection neutralizing abilities of sera from mice immunized with cocktail vaccines collected at different time points.

(A) Epithelial cell infection neutralizing titers of sera collected at week 1 from mice immunized with NP-cocktail, Free-cocktail and Al-cocktail.

(B) Epithelial cell infection neutralizing titers of sera collected at week 3 from mice immunized with NP-cocktail, Free-cocktail and Al-cocktail.

(C) Epithelial cell infection neutralizing titers of sera collected at week 5 from mice immunized with NP-cocktail, Free-cocktail and Al-cocktail.

(D) Epithelial cell infection neutralizing titers of sera collected at week 50 from mice immunized with NP-cocktail, Free-cocktail and Al-cocktail.

Half maximal inhibitory dilution fold (ID50) was calculated by sigmoid trend fitting. Data points are shown as the mean ± SEM (n=5). Source data are provided as a Source Data file.

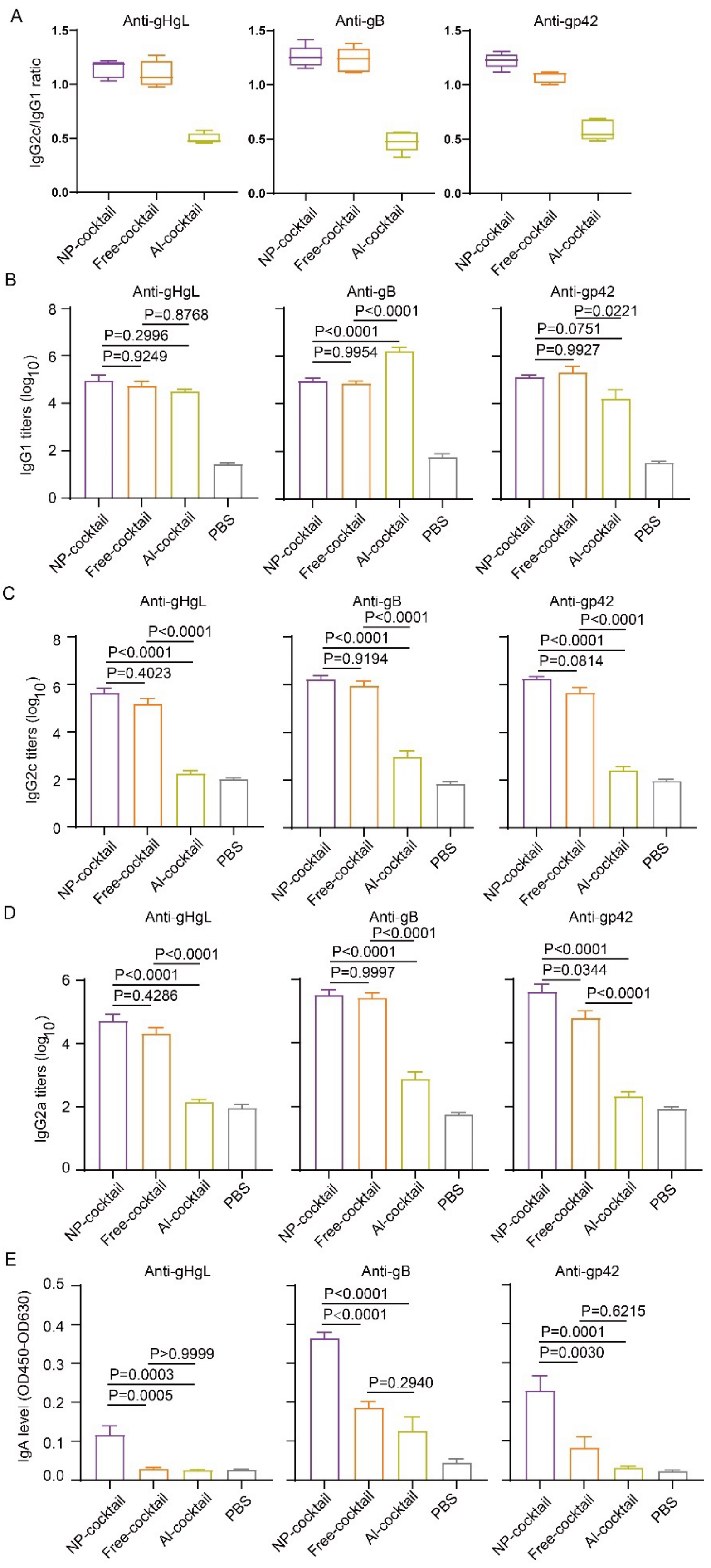

Figure S21. IgG subtypes and IgA levels induced by different cocktail vaccine formulations in C57BL/6J mice.

(A) IgG2c/IgG1 ratio of sera from C57BL/6J mice immunized with different vaccine formulations, on day 35. The center line indicates the median, upper and lower box lines show quartiles; and whiskers show the maximum and minimum values (n=5). (B-D) Anti-gHgL (left panel), anti-gB (middle panel) and anti-gp42 (right panel) IgG1 titers (B), IgG2c titers (C) and IgG2a titers (D) of sera from C57BL/6J mice immunized with different vaccine formulations, on day 35. (E) Serum IgA levels induced by different vaccine formulations, on day 35. (B-E) Data are shown as mean ± SEM (n=5). P values calculated using one-way ANOVA with Turkey's multiple comparison are shown as precise values. Source data are provided as a Source Data file.

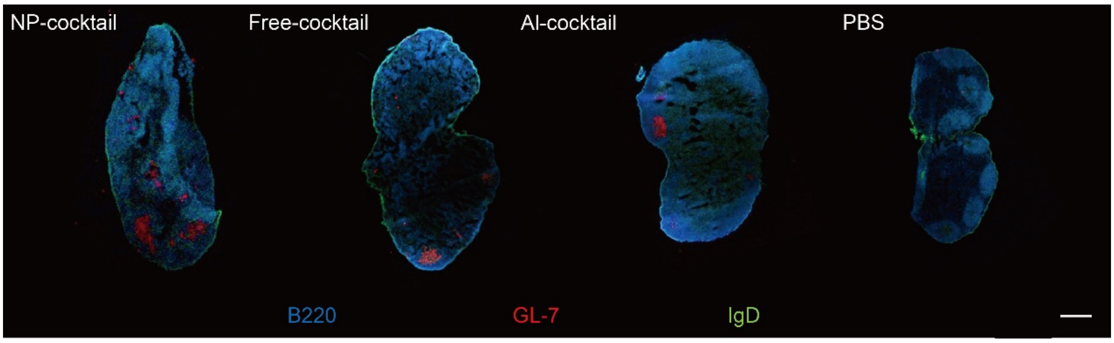

Figure S22. Germinal center formation in inguinal lymph nodes (LNs) of C57BL/6J mice immunized with the indicated vaccine formulations. LNs were collected on day 35 (Scale bar = 400 μm) (n=5).

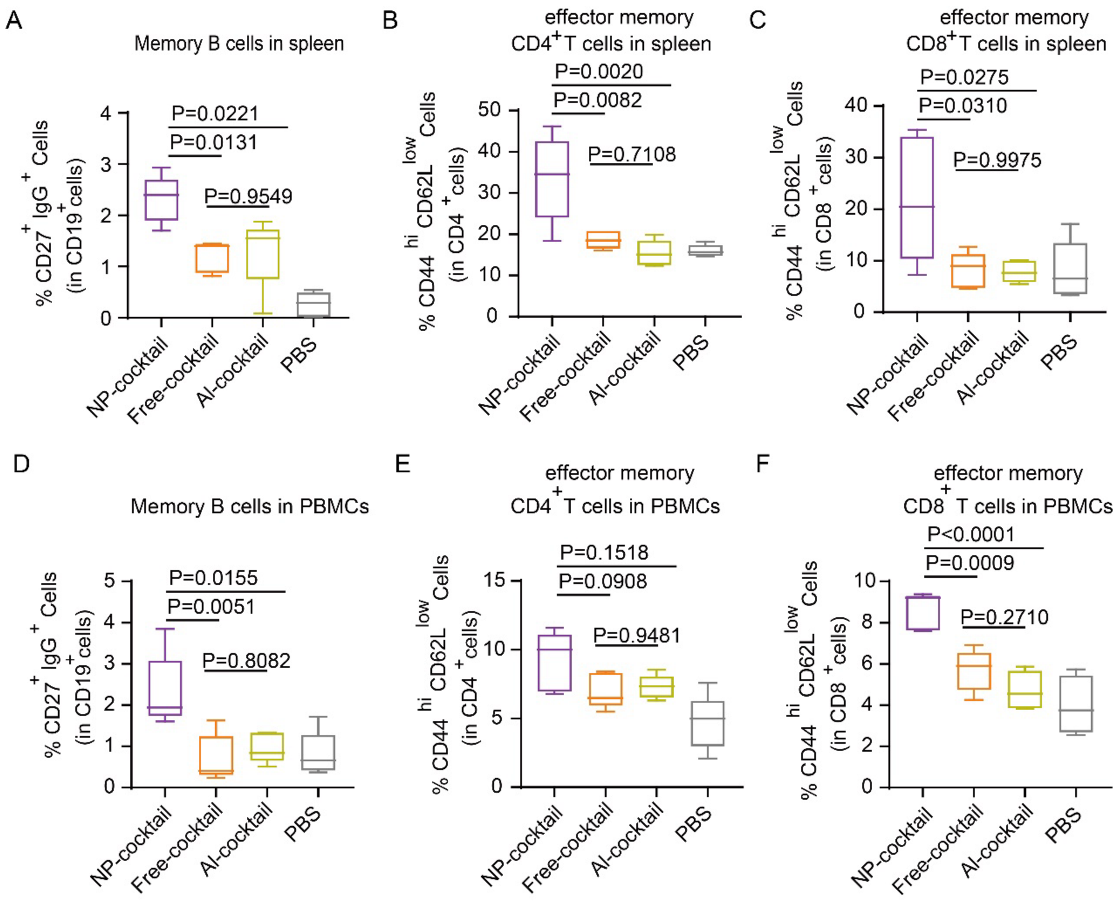

Figure S23. The nanoparticle cocktail vaccine enhances the production of memory T cells and B cells.

Splenocytes and PBMCs were analyzed by flow cytometry according to the strategy described in figure S7.

(A-C) Memory B cells (A), effector memory $CD4^+$ T cells (B) and $CD8^+$ T cells (C) in spleen were detected 1 week after the third immunization of C57BL/6J mice (day 35).

(D-F) Memory B cells (D), effector memory $CD4^+$ T cells (E) and $CD8^+$ T cells (F) in peripheral blood were detected 8 weeks after the third immunization of C57BL/6J mice (week 12).

(A-F) Data are shown as mean ± SEM (n=5). P values calculated using one-way ANOVA with Turkey's multiple comparison are shown as precise values. Source data are provided as a Source Data file. The center line indicates the median, upper and lower box lines show quartiles; and whiskers show the maximum and minimum values.

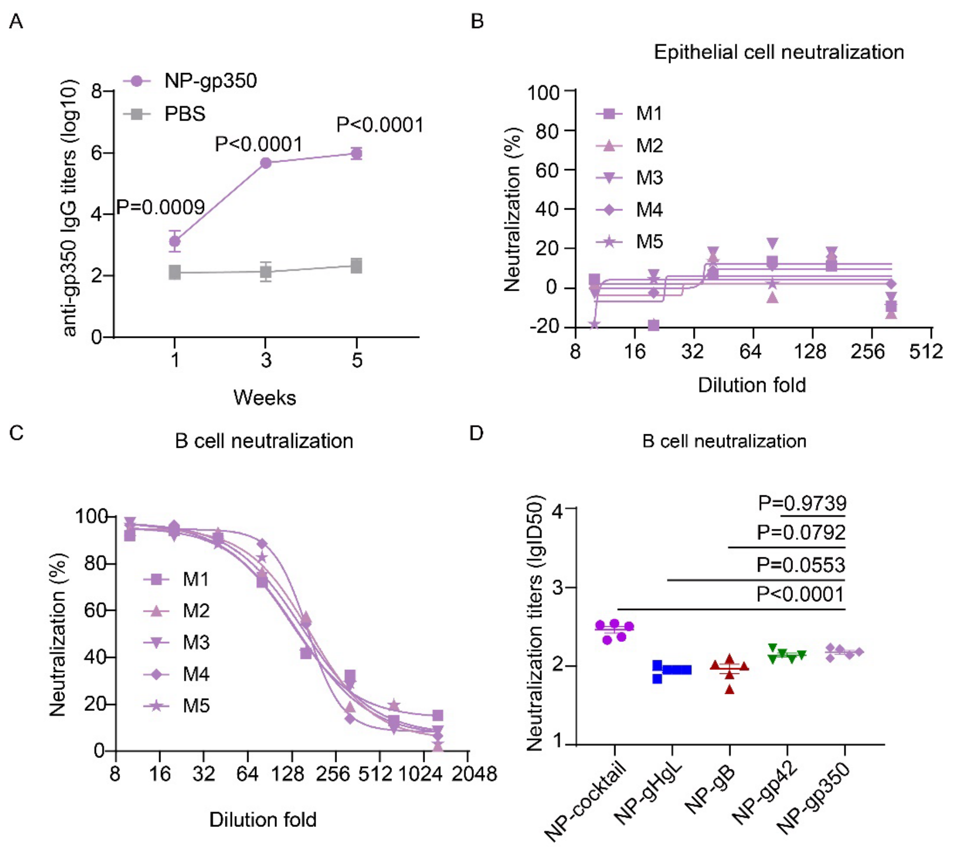

Figure S24. Immunogenicity evaluation of NP-gp350.

(A) The total IgG titers of sera collected from C57BL/6J mice immunized with NP-gp350. Data points are shown as the mean ± SEM (n=5). Statistical analysis was performed using unpaired welch's t test (n=5).

(B-C) Neutralization of Akata-EBV infection of HNE1 epithelial cells (B) and CNE2-EBV infection of Akata B cells (C) by individual sera collected on day 35 from five C57BL/6J mice immunized with NP-gp350 (M1-M5).

(D) Comparison of B cell neutralizing titers of sera collected on day 35 from C57BL/6J mice immunized with cocktail (NP-gHgL+NP-gB+NP-gp42) or individual nanovaccines. The indicated half maximal inhibitory dilution fold (ID50) values were calculated by sigmoid trend fitting. Data are shown as mean ID50 (n=5).

P values calculated using one-way ANOVA with Turkey's multiple comparison are shown as precise values. Source data are provided as a Source Data file.

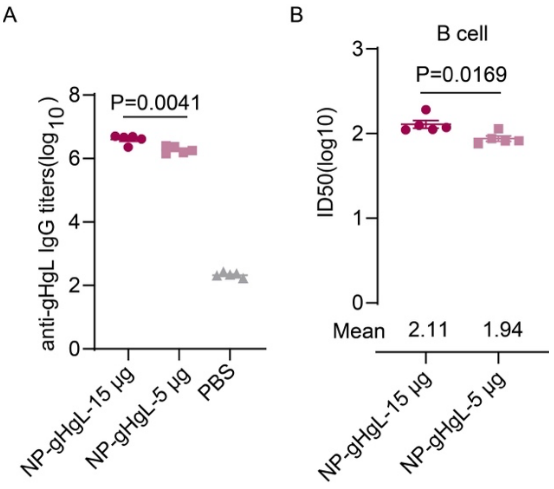

Figure S25. Dose-dependent antibody responses of NP-gHgL.

(A) Total IgG titers of sera collected on day 35 from C57BL/6J mice immunized with 15 µg or 5 µg NP-gHgL.

(B) Neutralization of CNE2-EBV infection of Akata B cells by sera collected on day 35 from C57BL/6J mice immunized with 15 µg or 5 µg NP-gHgL.

Data points are shown as the mean ± SEM (n=5). P values calculated using unpaired two-tailed Welch's t test are shown as precise values. Source data are provided as a Source Data file.

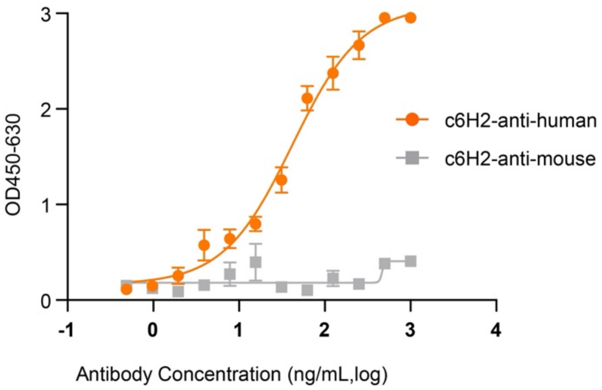

Figure S26. Reactivity of the chimeric 6H2 (c6H2) with anti-human or anti-mouse HRP conjugated secondary antibody (n=3). Source data are provided as a Source Data file.

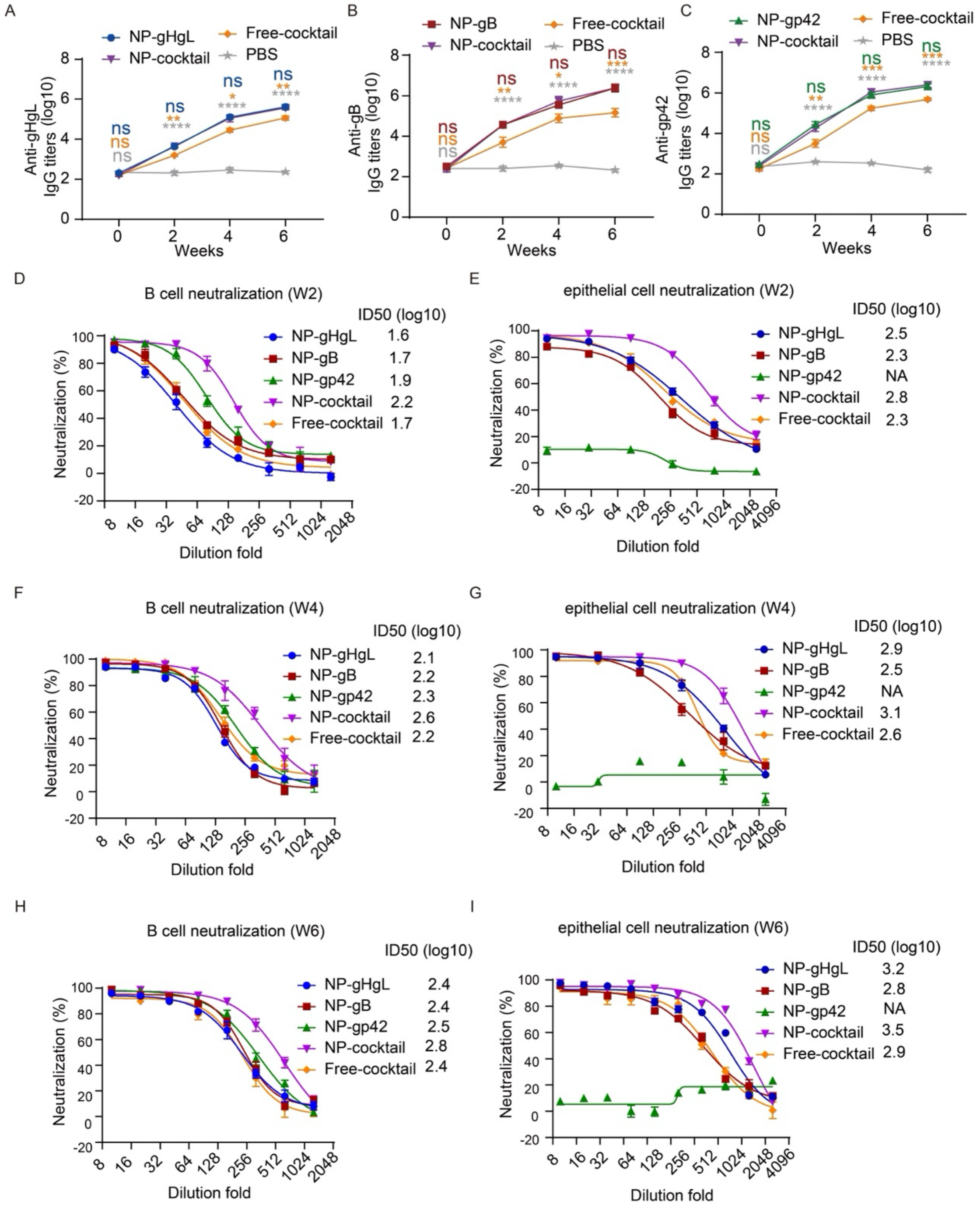

Figure S27. NP-cocktail induced robust humoral immune responses in rabbits. (A-C) IgG titers of sera collected on day 0, 14, 28 and 42 from rabbits immunized with different vaccine formulations. (A) Anti-gHgL IgG titers elicited by NP-gHgL, NP-cocktail, Free-cocktail and PBS, respectively. (B) Anti-gB IgG titers elicited by NP-gB, NP-cocktail, Free-cocktail and PBS, respectively. (C) Anti-gp42 IgG titers elicitied by NP-gp42, NP-cocktail, Free-cocktail and PBS, respectively. P values calculated using one-way ANOVA with Dunnett’s multiple comparison are shown as

precise values.

(D-E) B cell infection neutralizing titers (D) and epithelial cell neutralizing titers (E) of sera collected at week 2 from rabbits immunized with different vaccine formulations.

(F-G) B cell infection neutralizing titers (F) and epithelial cell neutralizing titers (G) of sera collected at week 4 from rabbits immunized with different vaccine formulations.

(H-I) B cell infection neutralizing titers (H) and epithelial cell neutralizing titers (I) of sera collected at week 6 from rabbits immunized with different vaccine formulations. Half maximal inhibitory dilution fold (ID50) was calculated by sigmoid trend fitting.

(A-I) Data points are shown as the mean ± SEM (n=5). Source data are provided as a Source Data file.

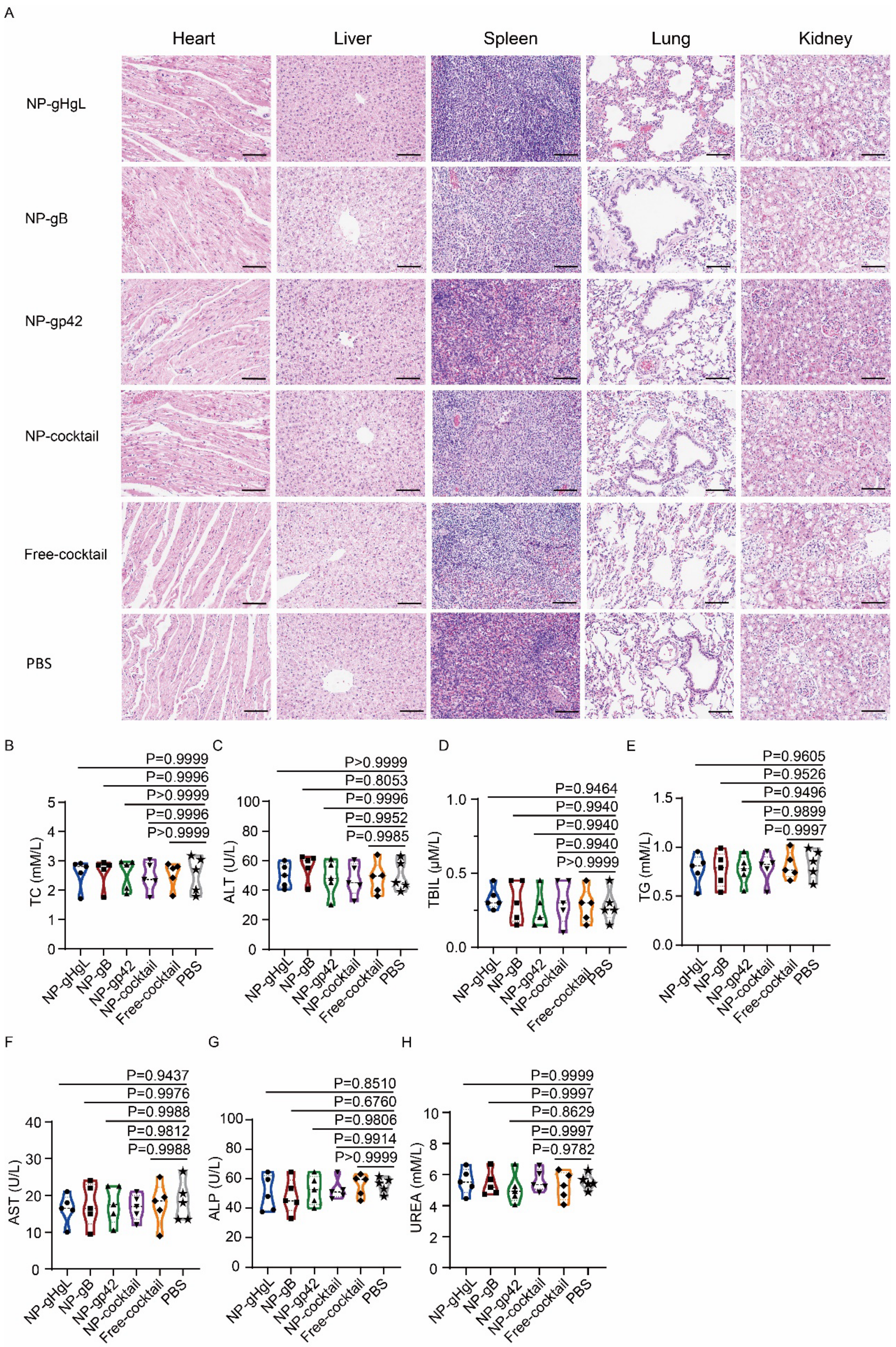

Figure S28. Safety of nanovaccines in rabbits.

(A) Representative sections of rabbit heart, liver, spleen, lung and kidney tissue stained by hematoxylin and eosin (HE) at necropsy 2 weeks after the third immunization (day 42) with the indicated vaccine formulations (n=5, dosage details in table S2) (Scale bar=50 μm) (n=5).

(B-H) Levels of TC (B), ALT (C), TBIL (D), TG (E), AST (F), ALP (G) and UREA (H) in the sera from rabbits immunized with various vaccines. Sera were collected at week 6 (n=5). P values calculated using one-way ANOVA with Dunnett's multiple comparison are shown as precise values. Source data are provided as a Source Data file.

total cholesterol, TC; alanine transaminase, ALT; total bilirubin, TBIL; triglycerides, TG; aspartate aminotransferase, AST; alkaline phosphatase, ALP.

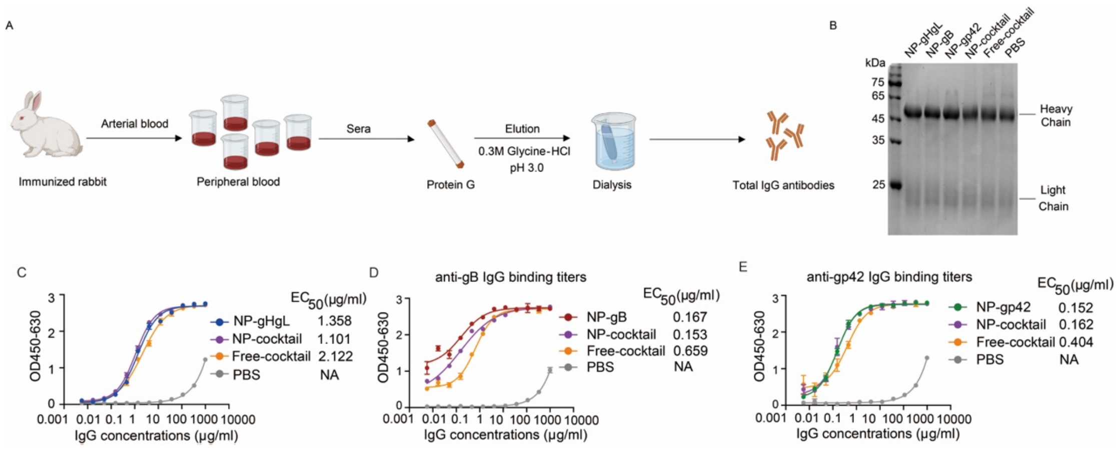

Figure S29. Total IgG antibodies purified from immunized rabbits.

(A) Schematic diagram of total IgG purification. The figure was created from Biorender.com.

(B) SDS-PAGE detection of purified total IgG antibodies (10 μg/lane). The gel was stained by Coomassie brilliant blue.

(C-E) The half-maximal effective concentration (EC50) of total IgG purified from rabbits that received different vaccines are shown. ELISA was performed using gHgL (C), gB (D) and gp42 (E) as target antigens to measure the specific binding of rabbit IgG from the different immunized groups. Data are shown as mean ± SEM of three independent replicates. Sera from three rabbits in the same treatment group were collected on day 42 and pooled prior to IgG purification.

Source data are provided as a Source Data file.

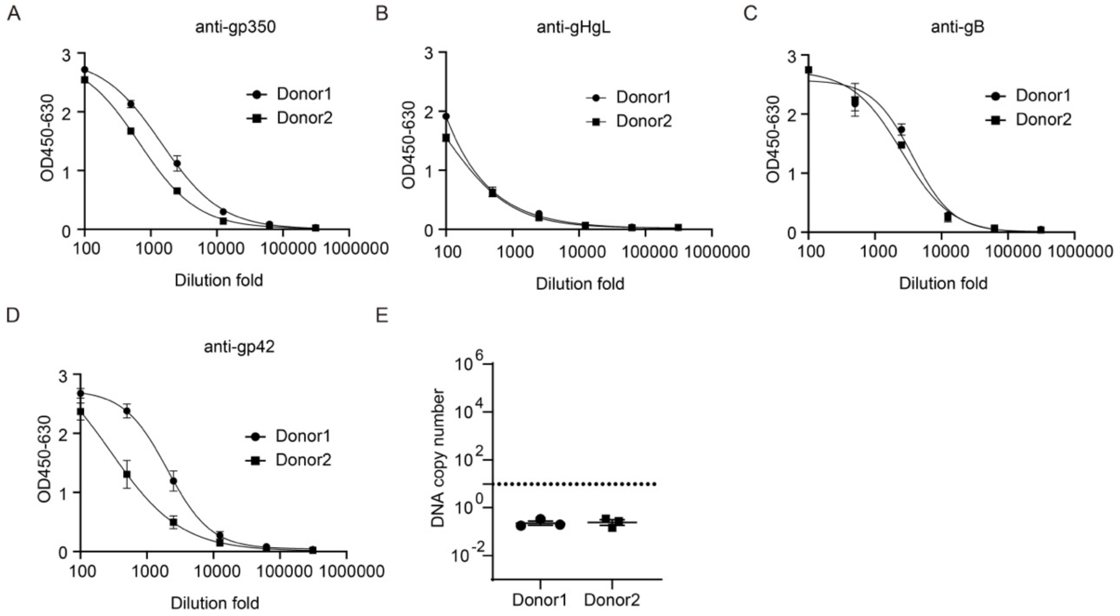

Figure S30. Human PBMCs used to reconstitute humanized mice.

(A-D) Anti-gp350 (A), anti-gHgL (B), anti-gB (C) and anti-gp42 (D) titers of sera from Donor1 and Donor2 determined by ELISA. Data are shown as mean ± SEM of three independent replicates.

(E) EBV DNA copy numbers of PBMCs isolated from Donor1 and Donor2. Data are shown as mean ± SEM of three independent replicates. The dashed line indicates the detection limit.

Source data are provided as a Source Data file.

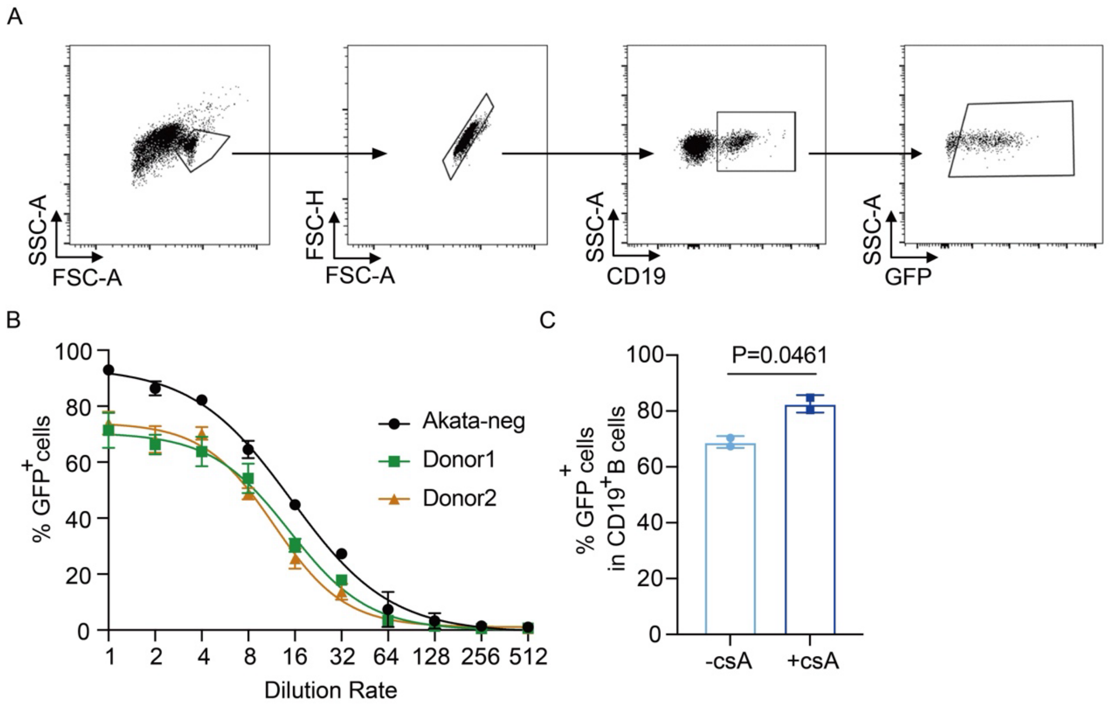

Figure S31. *In vitro* EBV infection of B cells in donors' PBMCs used for reconstitution of humanized mice.

(A) Gating strategies to analyze EBV infected B cells *in vitro*. 1×10^5 PBMCs from each donor were seeded in 96-well plate and serially diluted CNE2-EBV-GFP was added. After 48 h incubation, the percentage of GFP positive B cells was detected by flow cytometry. Cells without incubation with EBV were used as a negative control.

(B) Dose-dependent infection of B cells in donors' PBMCs infected by serially diluted CNE2-EBV-GFP virus (n=3). The gating strategy is shown in panel A. 1×10^5 Akata cells (EBV negative) infected by serial diluted CNE2-EBV-GFP were used as the positive control.

(C) Percentages of CNE2-EBV-GFP virus (1×) infected B cells in donors' PBMCs treated with/without Cyclosporine A (CsA). Data are shown as mean ± SEM (two donors). P values calculated using unpaired two-tailed Welch's t test are shown as precise values.

Source data are provided as a Source Data file.

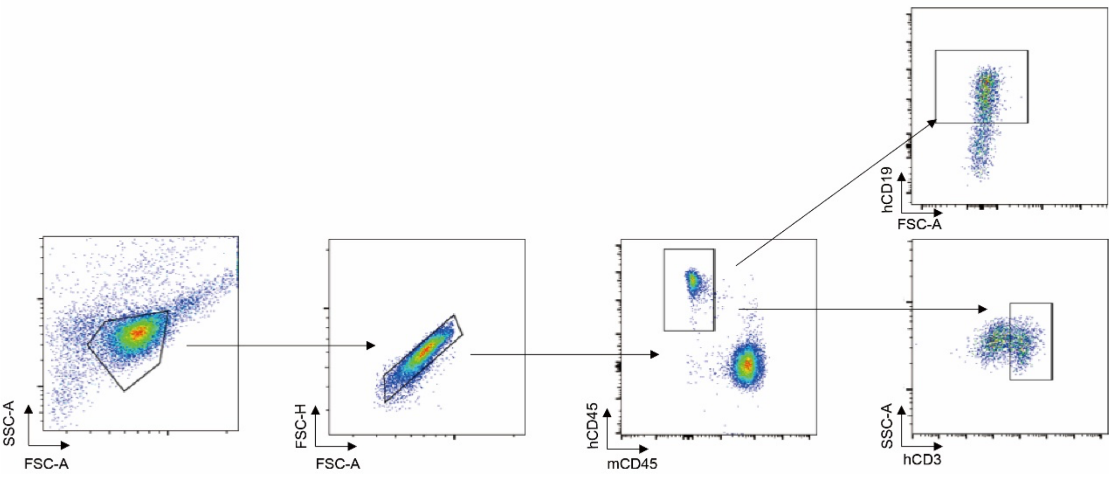

Figure S32. Representative flow cytometry plots showing the gating strategy used to analyze human immune cells in PBMCs, peritoneal lavage fluid, spleens, LNs or tumors collected from humanized mice (Figure 7D-7E, S33 and S36).

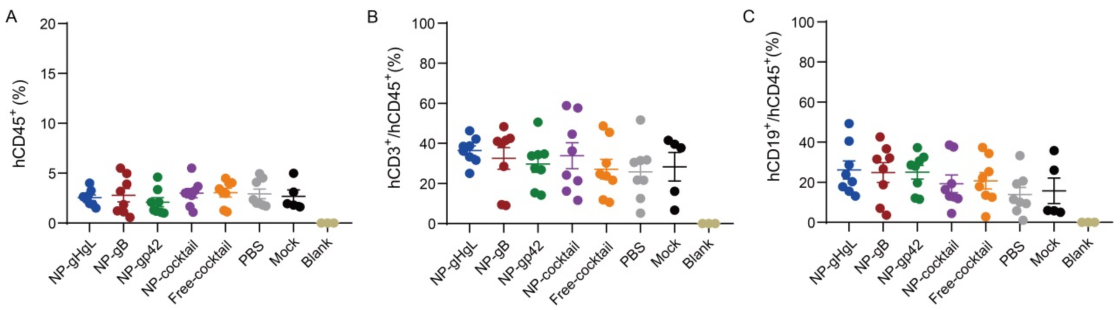

Figure S33. The percentage of human CD45^{+} cells, human CD3^{+} cells and human CD19^{+} cells in peripheral blood of humanized mice is shown. Mice were randomly assigned to groups to receive purified IgG from rabbits immunized with the indicated vaccine formulations. Blank mice were not engrafted with human PBMCs and served as negative control for detection by flow cytometry (detailed strategy in figure S18). All humanized mice were reconstituted by PBMCs from the same donor. Data are shown as mean ± SEM (Blank mice: n=3; humanized mice: n=53). Source data are provided as a Source Data file.

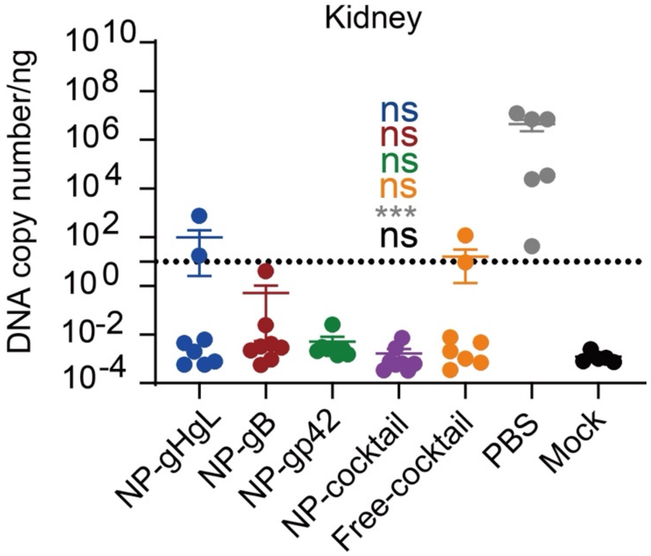

Figure S34. EBV DNA copy numbers in the kidneys of humanized mice treated with different IgG. The dashed line indicates the detection limit. Samples of two mice in the PBS group which died on day 7 and 10 were not collected because of decay. Data are shown as mean ± SEM (n=8 for the NP-gHgL, NP-gB, NP-gp42, NP-cocktail and Free-cocktail groups, n=6 for the PBS group and n=5 for the mock group). Statistical analysis was performed using one-way ANOVA with Dunnett’s multiple comparison and the P value were provided in the Source Data file. Source data are provided as a Source Data file.

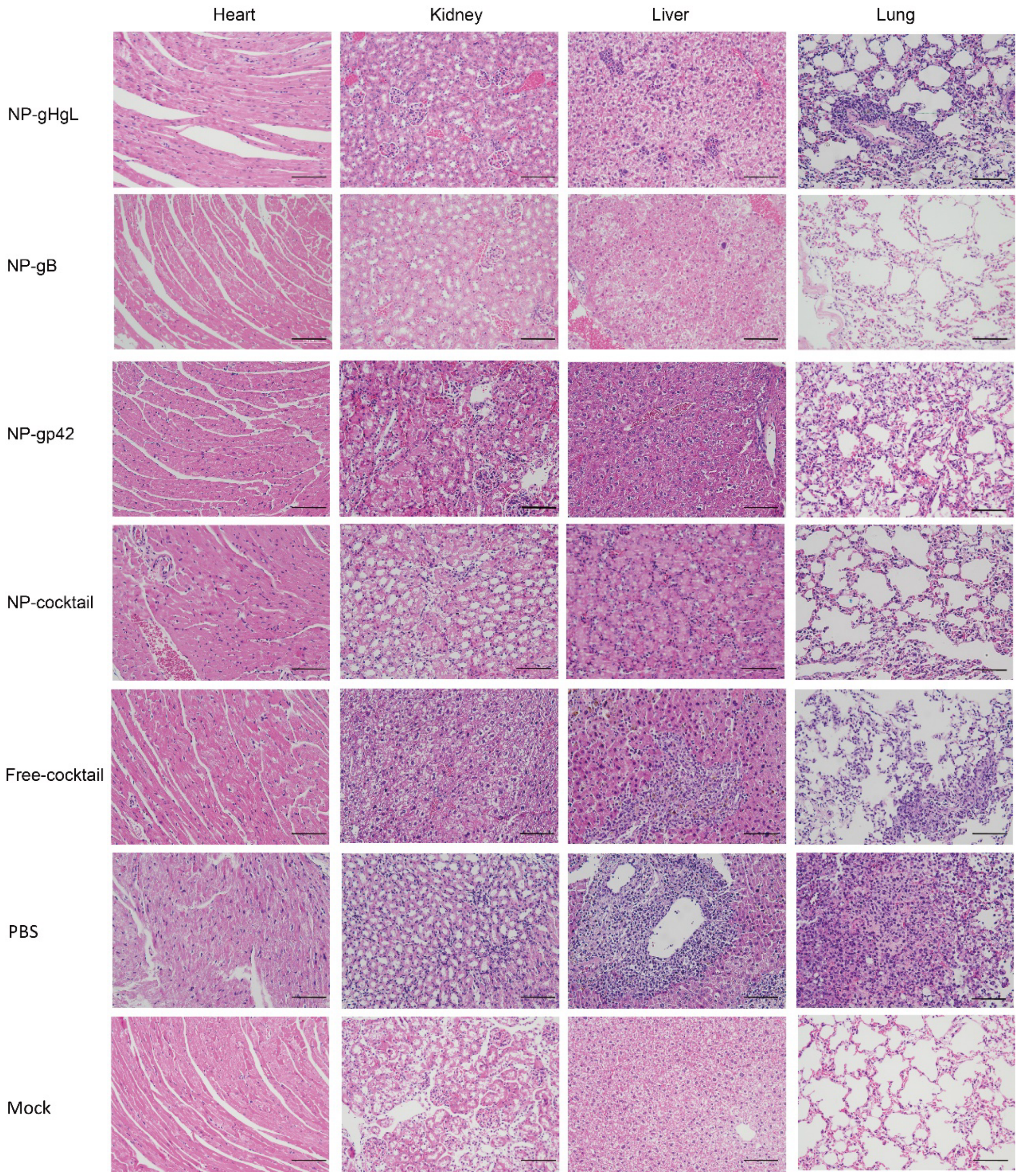

Figure S35. Representative sections of heart, liver, lung and kidney tissues stained for hematoxylin and eosin (HE) at necropsy of humanized mice treated with the IgG elicited by the indicated vaccine formulation and challenged with EBV. Mock mice were not treated nor challenged. (Scale bar=50 μm) (NP-gHgL, NP-gB, NP-gp42, NP-cocktail and Free-cocktail: n=8; PBS, n=6; Mock, n=5).

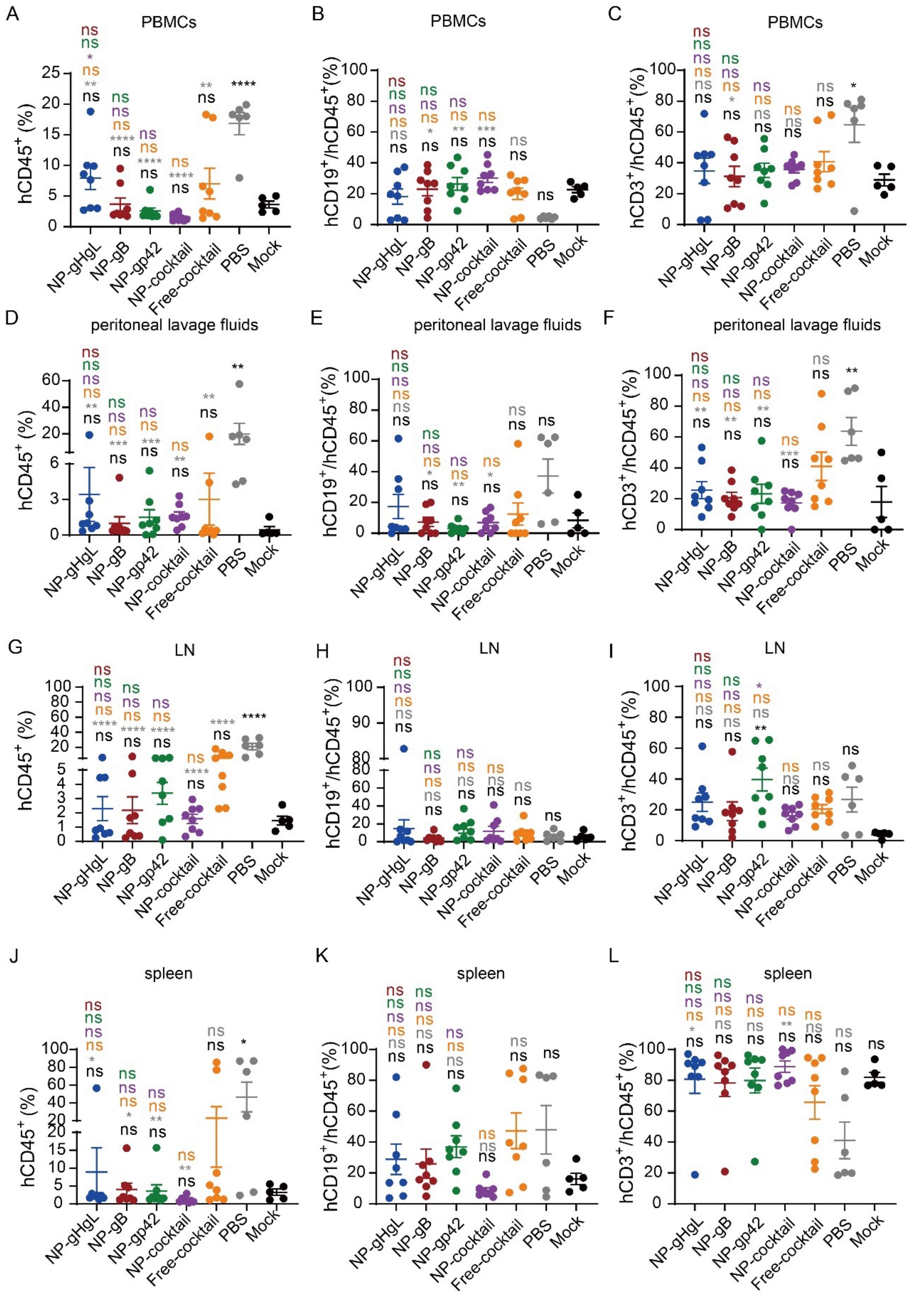

Figure S36. The percentage of human cells in humanized mice after different treatments and EBV challenge. Blood, peritoneal lavage fluid and tissues were collected from humanized mice at the end of the experiments (day 56) and analyzed by flow cytometry using the strategy described in figure S18.

(A-C) Percentages of human CD45$^+$ cells (A), human CD19$^+$ cells (B) and human CD3$^+$ cells (C) in the peripheral blood mononuclear cell (PBMCs) of humanized mice.

(D-F) Percentages of human CD45$^+$ cells (D), human CD19$^+$ cells (E) and human CD3$^+$ cells (F) in peritoneal lavage fluid of humanized mice.

(G-I) Percentages of human CD45$^+$ cells (G), human CD19$^+$ cells (H) and human CD3$^+$ cells (I) in mesenteric lymph nodes (LN) of humanized mice.

(J-L) Percentages of human CD45$^+$ cells (J), human CD19$^+$ cells (K) and human CD3$^+$ cells (L) in spleens of humanized mice.

Data are shown as mean ± SEM (NP-gHgL, NP-gB, NP-gp42, NP-cocktail and Free-cocktail: n=8; PBS, n=6; Mock, n=5). Statistical analysis was performed using one-way ANOVA with Turkey's multiple comparison and precise P values are shown in the Source data file. The color of the asterisks or ns denotes the statistical difference. Source data are provided as a Source Data file.

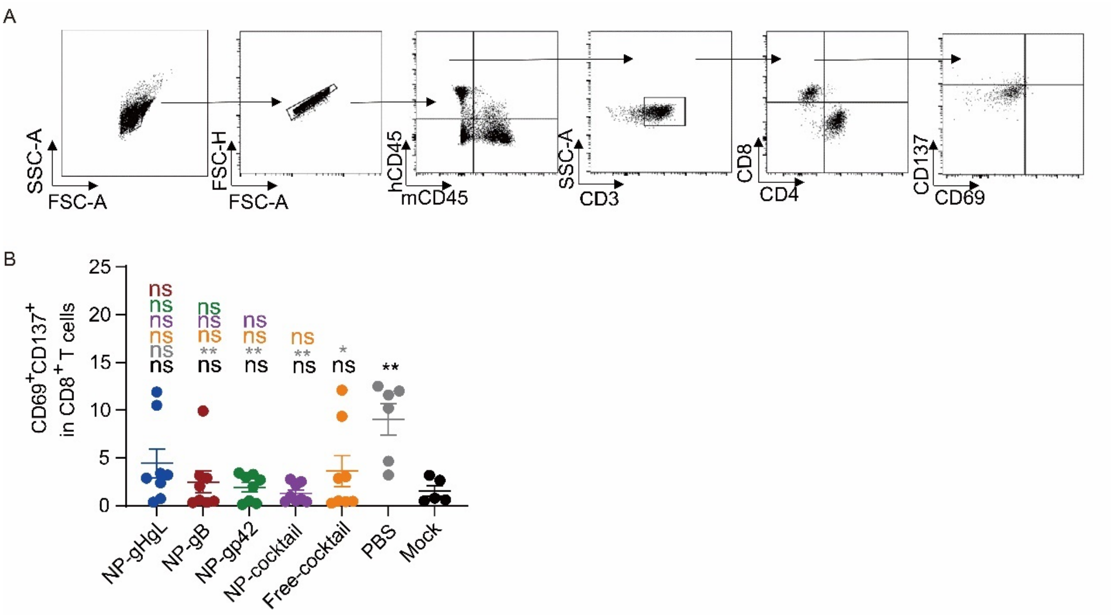

Figure S37. Gating strategy (A) and percentage (B) of hCD69^{+}hCD137^{+}hCD8^{+} T cells in humanized mice splenocytes. Data are shown as mean ± SEM (n=8 for the NP-gHgL, NP-gB, NP-gp42, NP-cocktail and Free-cocktail groups, n=6 for the PBS group and n=5 for the mock group). Statistical analysis was performed using one-way ANOVA with Turkey's multiple comparison and precise P values are shown in the Source data file. The color of the asterisks or ns denotes the statistical difference. Source data are provided as a Source Data file.

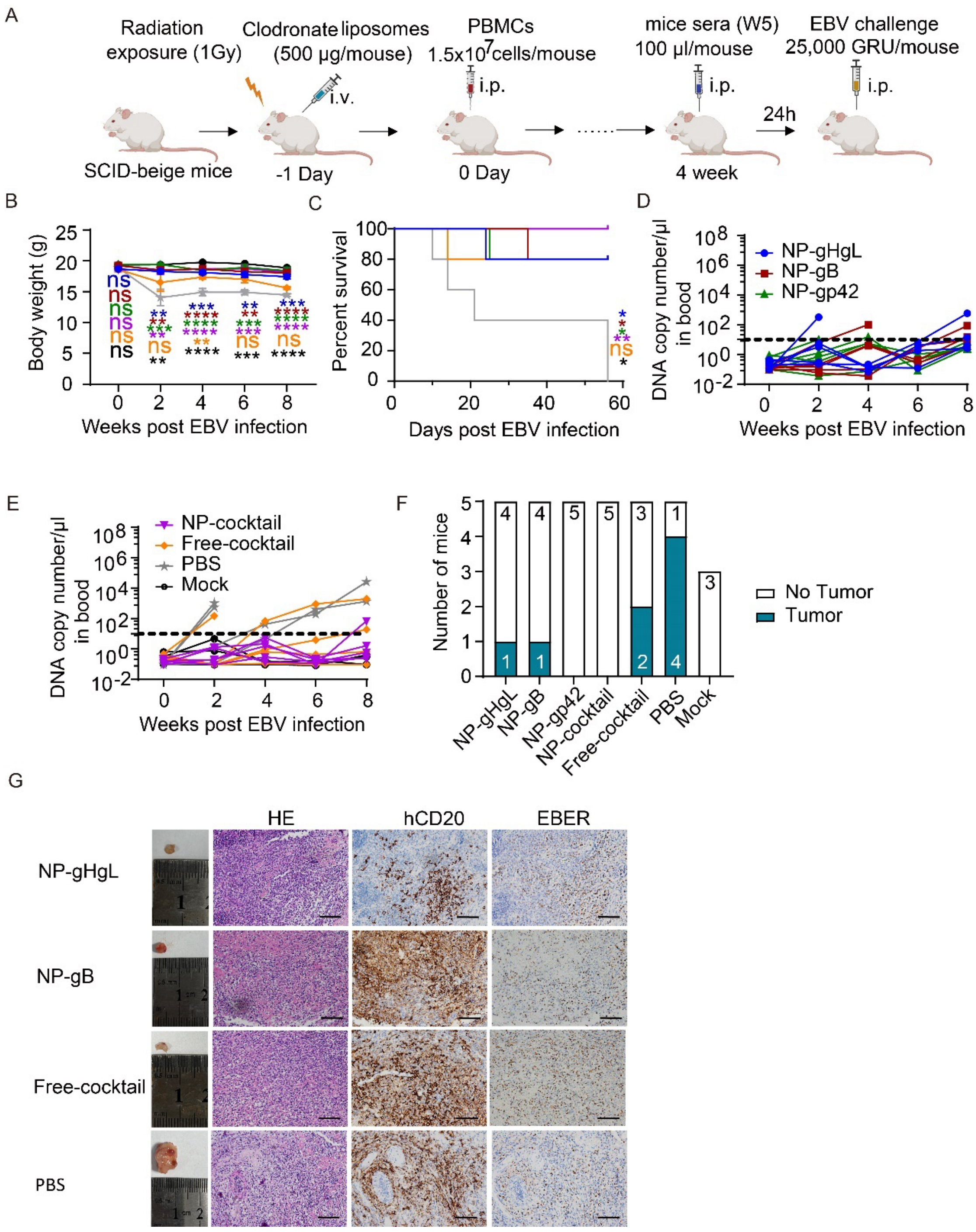

Figure S38. Sera collected at week 5 from mice immunized with NP-cocktail protected humanized mice from EBV associated lymphoma.

(A) Schematic illustration of humanized mice reconstitution and passive protection against EBV lethal challenge. SCID-beige mice were irradiated and administrated with clodronate liposomes before being reconstituted with PBMCs from healthy donors (EBV seropositive). Four weeks later, humanized mice were inoculated with sera of mice immunized with different vaccines. 25,000 GRU (green Raji units) Akata

strain EBV was injected 24 hours after mice sera administration. i.v., intravenously; i.p., intraperitoneally. The figure was created from Biorender.com. Humanized mice in the mock group were not challenged with EBV.

(B) Body weight of mice post-EBV challenge. Data are shown as mean ± SEM. Statistical analysis was performed using one-way ANOVA with Dunnett's multiple comparison and precise P values are shown in the Source data file. The color of the asterisks or ns denotes the statistical difference.

(C) Survival plot of humanized mice after EBV challenge. P values are shown in the Source data file. The color of the asterisks or ns denotes the statistical difference.

(D-E) EBV DNA copy numbers in peripheral blood of each group. Each line represents an individual mouse and the dashed line indicates the detection limit.

(F) Numbers of humanized mice with different treatment developed EBV-associated lymphoma.

(G) Representative macroscopic tumors and tumor tissues (n=1 for NP-gHgL, NP-gB; n=2 for Free-cocktail; n=4 for PBS) stained by HE, hybridized for EBER, and immunostained for $hCD20^{+}$ at necropsy (Scale bar=50 μm).

Source data are provided as a Source Data file.

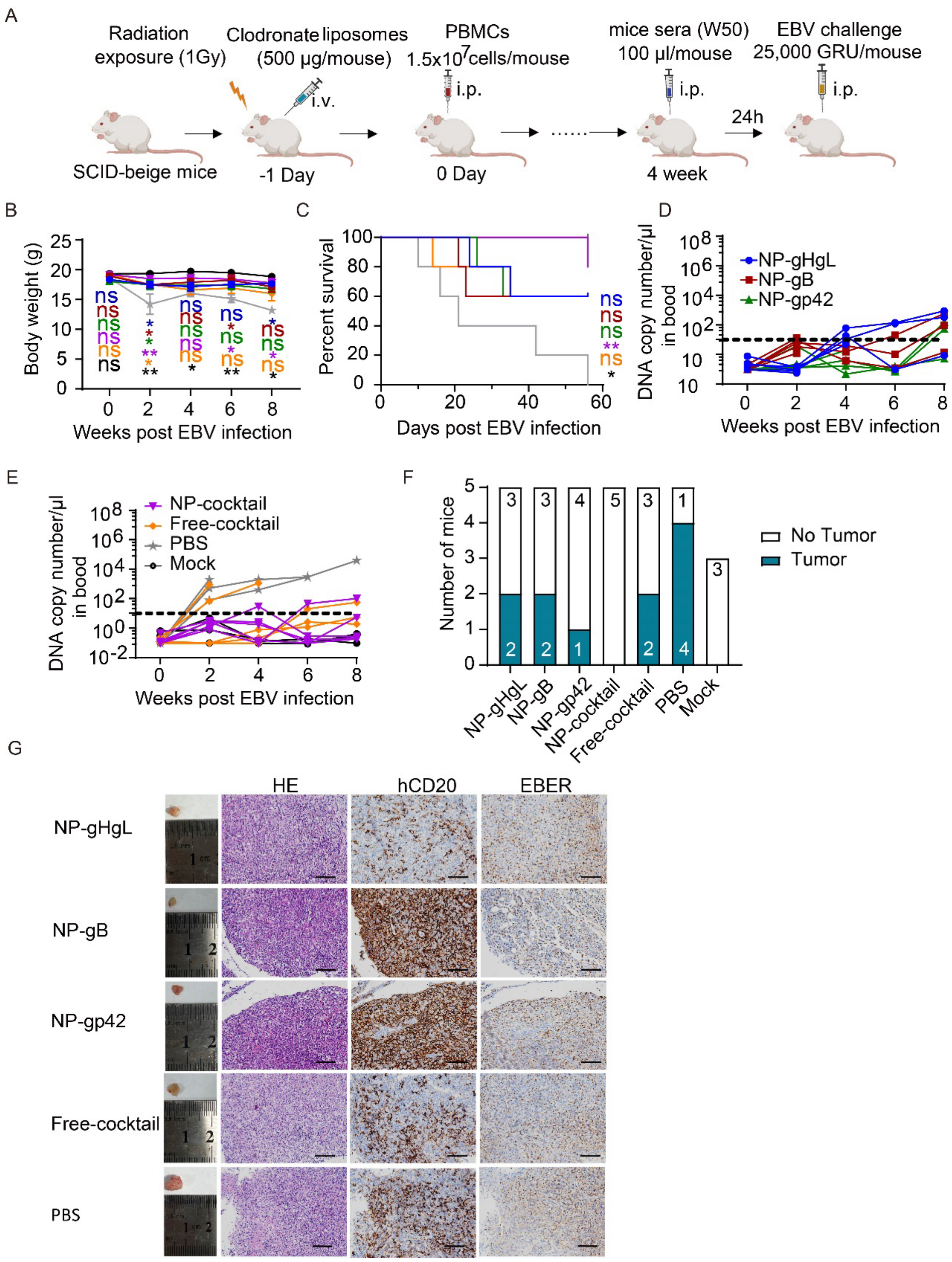

Figure S39. Sera collected at week 50 from mice immunized with NP-cocktail protected humanized mice from EBV associated lymphoma.

(A) Schematic illustration of humanized mice reconstitution and passive protection against EBV lethal challenge. SCID-beige mice were irradiated and administrated with clodronate liposomes before being reconstituted with PBMCs from healthy donors (EBV seropositive). Four weeks later, humanized mice were inoculated with

sera from mice immunized with different vaccines. 25,000 GRU (green Raji units) Akata strain EBV was injected 24 hours after mice sera administration. i.v., intravenously; i.p., intraperitoneally. The figure was created from Biorender.com. Humanized mice in the mock group were not challenged with EBV.

(B) Body weight of mice post-EBV challenge. Data are shown as mean ± SEM. Statistical analysis was performed using one-way ANOVA with Dunnett's multiple comparison and precise P values are shown in the Source data file. The color of the asterisks or ns denotes the statistical difference.

(C) Survival plot of humanized mice after EBV challenge. P values are shown in the Source data file. The color of the asterisks or ns denotes the statistical difference.

(D-E) EBV DNA copy numbers in peripheral blood of each group. Each line represents an individual mouse and the dashed line indicates the detection limit.

(F) Numbers of humanized mice with different treatment developed EBV-associated lymphoma.

(G) Representative macroscopic tumor and tumor tissue (n=2 for NP-gHgL, NP-gB and Free-cocktail; n=1 for NP-gp42; n=4 for PBS) stained by HE, hybridized for EBER, and immunostained for hCD20^{+} at necropsy (Scale bar=50 μm).

Source data are provided as a Source Data file.

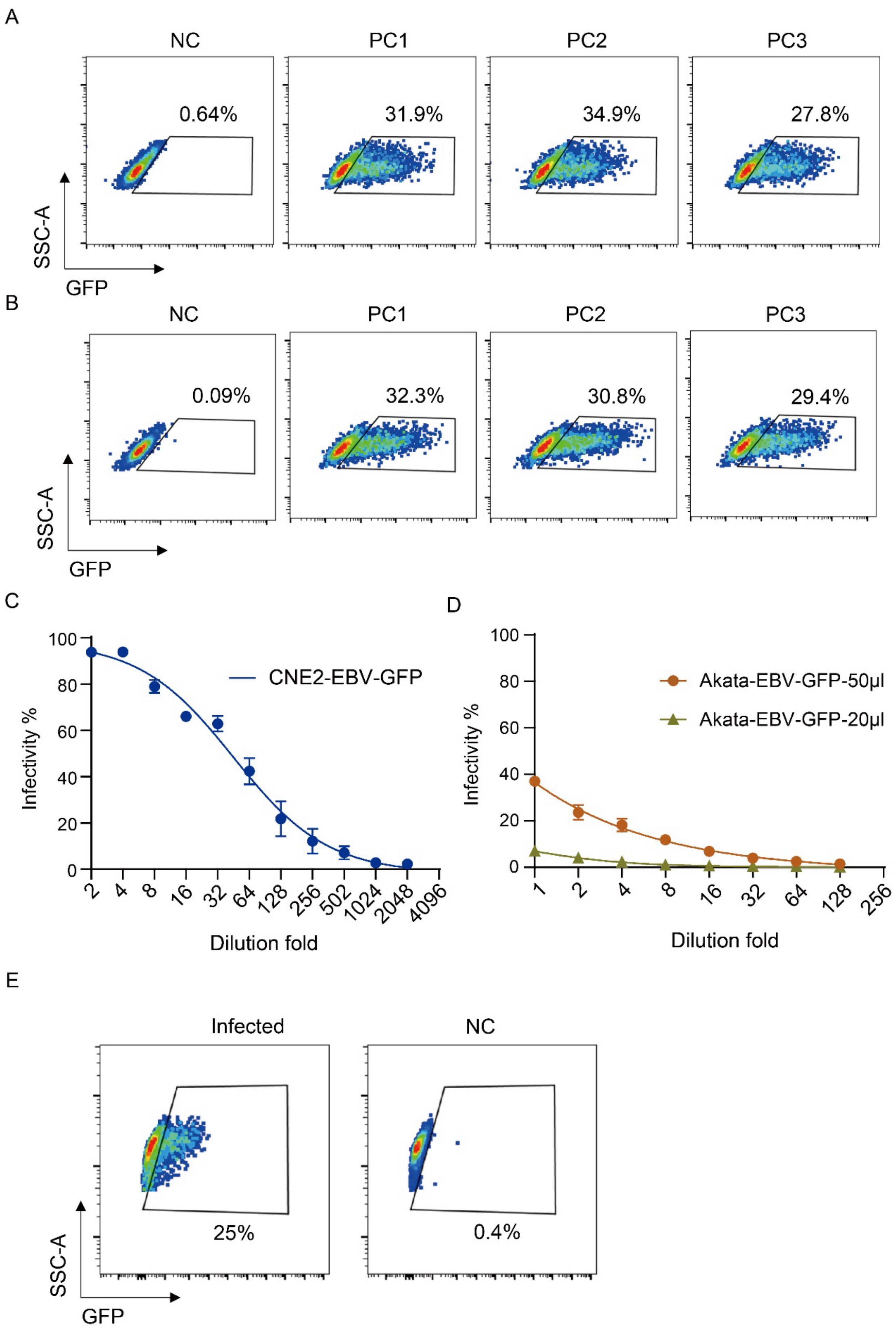

Figure S40. Titration of virus stocks used in neutralization assays and infections of humanized mice.

(A-B) Percentage of infected cells (GFP-positive) achieved in the absence of

antibodies/serum for epithelial cell neutralization experiment (A) and B cell neutralizing experiment (B). NC represents cells did not incubated with virus and PC1-PC3 represents three independent replicates of positive control.
(C-D) Infectivity titration curves for CNE2-EBV-GFP virus on Akata cells (C) and Akata-EBV-GFP on HNE1 cells (D) used in neutralization assay. Data are shown as mean ± SEM of three independent replicates.
(E) GRU determination of the virus dose used in humanized mice assays.
Source data are provided as a Source Data file.

Supplementary table

Supplementary table 1. Demographic information of the blood donors for antibody depletion assay.

No.	Sex	Age
190001	F	50-59
190002	M	40-49
190003	M	50-59
190004	F	20-29
190005	F	20-29
190006	F	20-29
190007	F	20-29
190008	M	20-29
190009	M	40-49
190010	M	20-29
190011	F	20-29
190012	F	20-29
190013	M	50-59
190014	F	20-29
190015	F	20-29
190016	F	20-29
190017	F	20-29
190018	M	20-29
190019	F	20-29
190020	F	20-29
190021	F	20-29
190022	F	20-29
190023	M	30-39
190024	F	20-29

190025	M	20-29
190026	F	20-29
190027	M	20-29
190028	M	20-29
190029	F	40-49
190030	F	20-29
190031	F	20-29
190032	M	30-39

F: Female; M: Male

Supplementary table 2. Composition of different vaccine formulations

Animals	Vaccines	Antigens/injection	Adjuvants/injection
C57BL/6J	NP-gHgL	gHgL 5 µg	CpG 10 µg; MPLA 10 µg
	NP-gB	gB 5 µg	CpG 10 µg; MPLA 10 µg
	NP-gp42	gp42 5 µg	CpG 10 µg; MPLA 10 µg
	Free-gHgL	gHgL 5 µg	CpG 10 µg; MPLA 10 µg
	Free-gB	gB 5 µg	CpG 10 µg; MPLA 10 µg
	Free-gp42	gp42 5 µg	CpG 10 µg; MPLA 10 µg
	Al-gHgL	gHgL 5 µg	Alum 10 µg
	Al-gB	gB 5 µg	Alum 10 µg
	Al-gp42	gp42 5 µg	Alum 10 µg
	eNP	/	/
	eNP-C-M	/	CpG 10 µg; MPLA 10 µg
	NP-gp350	gp350 5 µg	CpG 10 µg; MPLA 10 µg
C57BL/6J	NP-cocktail	gHgL 5 µg + gB 5 µg + gB 5 µg	CpG 10 µg; MPLA 10 µg (Total dose/mouse)
	Free-cocktail	gHgL 5 µg + gB 5 µg + gB 5 µg	CpG 10 µg; MPLA 10 µg (Total dose/mouse)
	Al-cocktail	gHgL 5 µg + gB 5 µg + gB 5 µg	Alum 10 µg (Total dose/mouse)
C57BL/6J	NP-gHgL-15 µg	gHgL 15 µg	CpG 10 µg; MPLA 10 µg
	NP-HgL-5 µg	gHgL 5 µg	CpG 10 µg; MPLA 10 µg
Rabbit	NP-gHgL	gHgL 100 µg	CpG 100 µg; MPLA 100 µg
	NP-gB	gB 100 µg	CpG 100 µg; MPLA 100 µg
	NP-gp42	gp42 100 µg	CpG 100 µg; MPLA 100 µg
	NP-cocktail	gHgL 100 µg + gB 100 µg + gB 100 µg	CpG 100 µg; MPLA 100 µg (Total dose/rabbit)
	Free-cocktail	gHgL 100 µg + gB 100 µg + gB 100 µg	CpG 100 µg; MPLA 100 µg (Total dose/rabbit)

Supplementary table 3. ID50 and ID90 of the sera from immunized animals

Infection model	Animal	Group	ID50	ID90
B cell infection neutralization	C57BL/6J	NP-gHgL	88.93	8.25
		Free-gHgL	49.94	NA
		Al-gHgL	29.71	2.287
		NP-gB	92.70	9.52
		Free-gB	36.88	NA
		Al-gB	36.41	5.32
		NP-gp42	137.90	50.37
		Free-gp42	88.02	27.74
		Al-gp42	35.38	NA
		NP-cocktail	290.6	91.55
		Free-cocktail	104.2	27.71
		Al-cocktail	60.59	15.18
Epithelial cell infection neutralization	C57BL/6J	NP-gHgL	853.60	217.11
		Free-gHgL	183.70	25.90
		Al-gHgL	76.90	NA
		NP-gB	516.0	121.84
		Free-gB	136.4	21.96
		Al-gB	54.59	NA
		NP-gp42	NA	NA
		Free-gp42	NA	NA
		Al-gp42	NA	NA
		NP-cocktail	2674.60	711.61
		Free-cocktail	996.40	128.90
		Al-cocktail	498.00	17.44
B cell infection neutralization	New Zealand white rabbits	NP-gHgL	226.00	49.27
		NP-gB	250.10	86.15
		NP-gp42	386.00	82.00

		NP-cocktail	850.80	143.67
		Free-cocktail	242.00	49.25
Epithelial cell infection neutralization	New Zealand white rabbits	NP-gHgL	1688	193.37
		NP-gB	791.7	47.23
		NP-gp42	NA	NA
		NP-cocktail	2902	467.32
		Free-cocktail	881.6	54.65

The sera were diluted from 1:10 and ID50 as well as ID90 values were calculated by sigmoid trend fitting using GraphPad Prism 8.0. NA means the value can not be calculated.

Supplementary table 4. PBMCs from different donors for humanized mice reconstitution.

Donor	Numbers of reconstituted SCID-beige mice	Injection agents
Donor1	8	1 mg IgG from NP-gHgL immunized rabbit
	8	1 mg IgG from NP-gB immunized rabbit
	8	1 mg IgG from NP-gp42 immunized rabbit
	8	1 mg IgG from NP-cocktail immunized rabbit
	8	1 mg IgG from Free-cocktail immunized rabbit
	8	1 mg IgG from PBS administrated rabbit
	5	Mock1
Donor2	5	Sera (week5) from NP-gHgL immunized mice
	5	Sera (week5) from NP-gB immunized mice
	5	Sera (week5) from NP-gp42 immunized mice
	5	Sera (week5) from NP-cocktail immunized mice
	5	Sera (week5) from Free-cocktail immunized mice
	5	Sera (week5) from PBS immunized mice
	5	Sera (week50) from NP-gHgL immunized mice
	5	Sera (week50) from NP-gB immunized mice
	5	Sera (week50) from NP-gp42 immunized mice
	5	Sera (week50) from NP-cocktail immunized mice
	5	Sera (week50) from Free-cocktail immunized mice
	5	Sera (week50) from PBS immunized mice
	3	Mock2

Approximate 1×10^9 PBMCs can be isolated from ~400 cc blood of one donor, which can be used to reconstitute ~66 SCID-beige mice. Hence, to avoid donor variability within each experiment, we reconstituted the humanized mice by PBMCs from 2 donors.

Reference

1. Hong J, *et al.* Antibody Generation and Immunogenicity Analysis of EBV gp42 N-Terminal Region. *Viruses* **13**, (2021).
