# Supplementary material for: A cocktail nanovaccine targeting key entry glycoproteins elicits high neutralizing antibody levels against EBV infection
Source: Nat Commun. 2024 Jun 21;15:5310. doi: 10.1038/s41467-024-49546-w (PMC11192767; doi:10.1038/s41467-024-49546-w)
Supplement: Supplementary file 3 — Reporting Summary [file 41467_2024_49546_MOESM3_ESM.pdf]

nature portfolio

Corresponding author(s): Claude Krummenacher Yi-Xin Zeng, Yongming Chen, Miao Xu, Xiao Zhang

Last updated by author(s): May 30, 2024

Reporting Summary

Nature Portfolio wishes to improve the reproducibility of the work that we publish. This form provides structure for consistency and transparency in reporting. For further information on Nature Portfolio policies, see our Editorial Policies and the Editorial Policy Checklist.

Statistics

For all statistical analyses, confirm that the following items are present in the figure legend, table legend, main text, or Methods section.

n/a	Confirmed	
☐	☒	The exact sample size (n) for each experimental group/condition, given as a discrete number and unit of measurement
☐	☒	A statement on whether measurements were taken from distinct samples or whether the same sample was measured repeatedly
☐	☒	The statistical test(s) used AND whether they are one- or two-sided<br>*Only common tests should be described solely by name; describe more complex techniques in the Methods section.*
☒	☐	A description of all covariates tested
☐	☒	A description of any assumptions or corrections, such as tests of normality and adjustment for multiple comparisons
☐	☒	A full description of the statistical parameters including central tendency (e.g. means) or other basic estimates (e.g. regression coefficient) AND variation (e.g. standard deviation) or associated estimates of uncertainty (e.g. confidence intervals)
☐	☒	For null hypothesis testing, the test statistic (e.g. F, t, r) with confidence intervals, effect sizes, degrees of freedom and P value noted<br>*Give P values as exact values whenever suitable.*
☒	☐	For Bayesian analysis, information on the choice of priors and Markov chain Monte Carlo settings
☒	☐	For hierarchical and complex designs, identification of the appropriate level for tests and full reporting of outcomes
☒	☐	Estimates of effect sizes (e.g. Cohen's d, Pearson's r), indicating how they were calculated

Our web collection on statistics for biologists contains articles on many of the points above.

Software and code

Policy information about availability of computer code

Data collection	IVIS Spectrum In Vivo Imaging System<br>Zetasizer Nano ZS<br>Tecnai T12 instrument<br>LSM 980 confocal microscope<br>CytoFLEX LX Flow Cytometer<br>ECLIPSE Ni-U microscopy<br>Bio-Tek EPOCH microplate reader
Data analysis	FlowJo software X 10.0.7<br>GraphPad Prism 8.0

For manuscripts utilizing custom algorithms or software that are central to the research but not yet described in published literature, software must be made available to editors and reviewers. We strongly encourage code deposition in a community repository (e.g. GitHub). See the Nature Portfolio guidelines for submitting code & software for further information.

Data

Policy information about availability of data

All manuscripts must include a data availability statement. This statement should provide the following information, where applicable:

- Accession codes, unique identifiers, or web links for publicly available datasets
- A description of any restrictions on data availability
- For clinical datasets or third party data, please ensure that the statement adheres to our policy

All data supporting the findings of this study are provided in the Supplementary Information/Source Data file. Source data are provided with this paper.

Research involving human participants, their data, or biological material

Policy information about studies with human participants or human data. See also policy information about sex, gender (identity/presentation), and sexual orientation and race, ethnicity and racism.

Reporting on sex and gender	Sex and gender based analysis is not relevant for the study.
Reporting on race, ethnicity, or other socially relevant groupings	Analysis of race, ethnicity, and other social groupings are not relevant for this study.
Population characteristics	Healthy volunteers who were willing to offer blood were recruited. No additional information was collected as requested by the ethical review committee for the purposes of this translational work.
Recruitment	All healthy volunteers recruited in the study offered blood samples and gave written informed consent. No self-selection bias was involved in this study. The only criteria used for recruitment of sera samples was EBV seropositivity.
Ethics oversight	The study was approved by Institutional Ethics Committee of the Sun Yat-sen University Cancer Center, Guangdong, China

Note that full information on the approval of the study protocol must also be provided in the manuscript.

Field-specific reporting

Please select the one below that is the best fit for your research. If you are not sure, read the appropriate sections before making your selection.

☒ Life sciences ☐ Behavioural & social sciences ☐ Ecological, evolutionary & environmental sciences

For a reference copy of the document with all sections, see nature.com/documents/nr-reporting-summary-flat.pdf

Life sciences study design

All studies must disclose on these points even when the disclosure is negative.

Sample size	Sample/group sizes ≥ 3 were chosen to support statistical analysis and variation expected based on similar published experiments.
Data exclusions	No data were excluded from the analyses.
Replication	All assays included in the manuscript were run at least three times and all included successful technical replicates. Further details are included in the manuscript.
Randomization	All mice, humanized mice and rabbit were allocated randomly.
Blinding	Study animals were purchased and randomly assigned to experimental groups. Investigators were not blinded during data collection for this study because we need to analyze which group behave better and why.

Reporting for specific materials, systems and methods

We require information from authors about some types of materials, experimental systems and methods used in many studies. Here, indicate whether each material, system or method listed is relevant to your study. If you are not sure if a list item applies to your research, read the appropriate section before selecting a response.

Materials & experimental systems

n/a	Involved in the study
	[x] Antibodies
	[x] Eukaryotic cell lines
[x]	Palaeontology and archaeology
	[x] Animals and other organisms
[x]	Clinical data
[x]	Dual use research of concern
[x]	Plants

Methods

n/a	Involved in the study
[x]	ChIP-seq
	[x] Flow cytometry
[x]	MRI-based neuroimaging

Antibodies

Antibodies used

Antibody / Source / Catalogue number / clone number / Dilution
Alexa Fluor 594 Anti-Mouse B220 BioLegend 103254 RA3-6B2 1:100
FITC Anti-Mouse CD3 BioLegend 100204 17A2 1:100
Monoclonal Anti-Mouse CD16/CD32 Antibody eBioscience 14-0161-82 93 1:100
Brilliant Violet 605 Anti-Mouse CD11c BioLegend 117333 N418 1:100
PE Cyanine7 Anti-Mouse CD86 BioLegend 105014 GL-1 1:100
PE Anti-Mouse CD80 BioLegend 104707 16-10A1 1:100
PerCP Cyanine5.5 Anti-Mouse I-A/I-E BioLegend 107626 M5/114.15.2 1:100
Alexa Fluor 594 Anti-Mouse IgD BioLegend 405740 11-26c.2a 1:100
Alexa Fluor 488 Anti-Mouse B220 BioLegend 103225 RA3-6B2 1:100
Alexa Fluor 647 Anti-Mouse GL7 BioLegend 144605 GL7 1:100
APC Cyanine7 Anti-Mouse CD45 BioLegend 103116 30-F11 1:100
Alexa Fluor 700 Anti-Mouse CD4 BioLegend 100430 GK1.5 1:100
APC Anti-Mouse CD8a BioLegend 100712 53-6.7 1:100
PE Cyanine7 Anti-Mouse IFN-γ BioLegend 505826 XMG1.2 1:100
Brilliant Violet 421 Anti-Mouse TNF-α BioLegend 506327 MP6-XT22 1:100
PE Anti-Mouse IL-2 BioLegend 503808 JES6-5H4 1:100
Brilliant Violet 605 Anti-Mouse B220 BioLegend 103243 RA3-6B2 1:100
PerCP Cyanine5.5 Anti-Mouse IgG BioLegend 405314 Poly4053 1:100
Brilliant Violet 421 Anti-Mouse CD27 BioLegend 124223 LG.3A10 1:100
PE Cyanine7 Anti-Mouse CD44 BioLegend 103030 IM7 1:100
PE Anti-Mouse CD62L BioLegend 161204 W18021D 1:100
APC Cyanine7 Anti-Human CD45 BioLegend 368516 2D1 1:100
APC Anti-Human CD19 BioLegend 302212 HIB19 1:100
FITC Anti-Human CD3 BioLegend 317305 OKT3 1:100
Brilliant Violet 510 Anti-Mouse CD45 BioLegend 103138 30-F11 1:100
Brilliant Violet 421 Anti-Mouse CXCR5 BIolegend 145511 L138D7 1:100
PerCP/Cyanine5.5 Anti-mouse Bcl6 Biolegend 358507 7D1 1:100
Recombinant Anti-Human CD20 Abcam ab64088 SP32 1:200
Goat Anti-Human IgG-HRP Promega W4031 polyclone 1:5000
Goat Anti-Mouse IgG-HRP Promega W4021 polyclone 1:5000
Goat Anti-Rabbit IgG-HRP Promega W4011 polyclone 1:5000
Goat Anti-Mouse IgA-HRP Bethyl Laboratories A90-103P polyclone 1:5000
Goat Anti-Mouse IgG1-HRP Bethyl Laboratories A90-105P polyclone 1:5000
Goat Anti-Mouse IgG2a-HRP Bethyl Laboratories A90-107P polyclone 1:5000
Goat Anti-Mouse IgG2c-HRP Bethyl Laboratories A90-136P polyclone 1:5000

Validation

https://www.biolegend.com/en-us/products/alexa-fluor-594-anti-mouse-human-cd45r-b220-antibody-9620
https://www.biolegend.com/en-us/products/fitc-anti-mouse-cd3-antibody-45
https://www.thermofisher.cn/cn/zh/antibody/product/CD16-CD32-Antibody-clone-93-Monoclonal/14-0161-82
https://www.biolegend.com/en-us/products/brilliant-violet-605-anti-mouse-cd11c-antibody-7865
https://www.biolegend.com/en-us/products/pe-cyanine7-anti-mouse-cd86-antibody-3046
https://www.biolegend.com/en-us/products/pe-anti-mouse-cd80-antibody-43
https://www.biolegend.com/en-us/products/percp-cyanine5-5-anti-mouse-i-a-i-e-antibody-4282
https://www.biolegend.com/en-us/products/alexa-fluor-594-anti-mouse-igd-antibody-11764
https://www.biolegend.com/en-us/products/alexa-fluor-488-anti-mouse-human-cd45r-b220-antibody-2707
https://www.biolegend.com/en-us/products/alexa-fluor-647-anti-mouse-human-gl7-antigen-t-and-b-cell-activation-marker-antibody-8602
https://www.biolegend.com/en-us/products/apc-cyanine7-anti-mouse-cd45-antibody-2530
https://www.biolegend.com/en-us/products/alexa-fluor-700-anti-mouse-cd4-antibody-3385
https://www.biolegend.com/en-us/products/apc-anti-mouse-cd8a-antibody-150
https://www.biolegend.com/en-us/products/pe-cyanine7-anti-mouse-ifn-gamma-antibody-5865

https://www.biolegend.com/en-us/products/brilliant-violet-421-anti-mouse-tnf-alpha-antibody-7336
https://www.biolegend.com/en-us/products/pe-anti-mouse-il-2-antibody-954
https://www.biolegend.com/en-us/products/brilliant-violet-605-anti-mouse-human-cd45r-b220-antibody-7870
https://www.biolegend.com/en-us/products/percp-cyanine5-5-goat-anti-mouse-igg-minimal-x-reactivity-6951
https://www.biolegend.com/en-us/products/brilliant-violet-421-anti-mouse-rat-human-cd27-antibody-11720
https://www.biolegend.com/en-us/products/pe-cyanine7-anti-mouse-human-cd44-antibody-3932
https://www.biolegend.com/en-us/products/pe-anti-mouse-cd62l-antibody-19844
https://www.biolegend.com/en-us/products/apc-cyanine7-anti-human-cd45-antibody-12400
https://www.biolegend.com/en-us/products/apc-anti-human-cd19-antibody-715
https://www.biolegend.com/en-us/products/fitc-anti-human-cd3-antibody-3644
https://www.biolegend.com/en-us/products/brilliant-violet-510-anti-mouse-cd45-antibody-7995
https://www.biolegend.com/en-gb/products/brilliant-violet-421-anti-mouse-cd185-cxcr5-antibody-8553?GroupID=BLG11476
https://www.biolegend.com/en-us/products/percp-cyanine5-5-anti-human-mouse-bcl-6-antibody-12192?GroupID=BLG11635
https://www.abcam.cn/products/primary-antibodies/cd20-antibody-sp32-ab64088.html
https://www.promega.com.cn/products/protein-detection/primary-and-secondary-antibodies/anti-human-igg-h-and-l-hrp-conjugate
https://www.promega.com.cn/products/protein-detection/primary-and-secondary-antibodies/anti_mouse-igg-h-and-l-hrp-conjugate
https://www.promega.com.cn/products/protein-detection/primary-and-secondary-antibodies/anti-rabbit-igg-h-and-l-hrp-conjugate
https://www.biomol.com/products/antibodies/secondary-antibodies/general/anti-mouse-iga-hrp-conjugated-a90-103p
https://www.biomol.com/products/antibodies/secondary-antibodies/general/anti-mouse-igg1-hrp-conjugated-a90-105p
https://www.biomol.com/products/antibodies/secondary-antibodies/general/anti-mouse-igg2a-hrp-conjugated-a90-107p
https://www.biomol.com/products/antibodies/secondary-antibodies/general/anti-mouse-igg2c-hrp-conjugated-a90-136p

Eukaryotic cell lines

Policy information about cell lines and Sex and Gender in Research

Cell line source(s)	Freestyle™ 293F cells was purchased from Thermo Fisher Scientific (Cat3 R79007). Akata-EBV-GFP and CNE2-EBV-GFP cells derived from parental cell lines were cultured in the presence of G418 (700 µg/ml). BMDCs were induced from female C57BL/6J mice and cultured in this study. DC2.4, Akata, CHO-K1 and HNE1 cells were derived from parental cell lines.
Authentication	Cell line authentication was performed by morphology check and EBV infection test.
Mycoplasma contamination	All cell lines are free of mycoplasma.
Commonly misidentified lines (See ICLAC register)	According to the ICLAC database, Akata and CNE2 cells may be contaminated by HeLa cells. However, unlike Akata-EBV-GFP or CNE2-EBV-GFP cells, HeLa cells are resistant to EBV infection, which rules out the risk of cell line misidentification in our study.

Animals and other research organisms

Policy information about studies involving animals; ARRIVE guidelines recommended for reporting animal research, and Sex and Gender in Research

Laboratory animals	C57BL/6J Mice 4-6w female<br>SCID-beige mice 4w female<br>New Zealand white Rabbit 6 month old female
Wild animals	No wild animals were used in the study.
Reporting on sex	Although we don't have sex bias, we have used female mice/rabbit in the study because male mice/rabbit tend to fight. Due to aggressive nature of the male mice/rabbit, they have been excluded from the studies.
Field-collected samples	No field collected samples were used in the study.
Ethics oversight	All mice experiments were performed under protocols approved by the Sun Yat-sen University Cancer Center Animal Care and Use Committee. All rabbits experiment were performed under protocols approved by the Sun Yat-sen University Animal Care and Use Committee. According to approved guidelines, animals were humanely euthanized at the end of each experiment.

Note that full information on the approval of the study protocol must also be provided in the manuscript.

Plants

Seed stocks

Report on the source of all seed stocks or other plant material used. If applicable, state the seed stock centre and catalogue number. If plant specimens were collected from the field, describe the collection location, date and sampling procedures.

Novel plant genotypes

Describe the methods by which all novel plant genotypes were produced. This includes those generated by transgenic approaches, gene editing, chemical/radiation-based mutagenesis and hybridization. For transgenic lines, describe the transformation method, the number of independent lines analyzed and the generation upon which experiments were performed. For gene-edited lines, describe the editor used, the endogenous sequence targeted for editing, the targeting guide RNA sequence (if applicable) and how the editor was applied.

Authentication

Describe any authentication procedures for each seed stock used or novel genotype generated. Describe any experiments used to assess the effect of a mutation and, where applicable, how potential secondary effects (e.g. second site T-DNA insertions, mosiacism, off-target gene editing) were examined.

Flow Cytometry

Plots

Confirm that:

- [x] The axis labels state the marker and fluorochrome used (e.g. CD4-FITC).
- [x] The axis scales are clearly visible. Include numbers along axes only for bottom left plot of group (a 'group' is an analysis of identical markers).
- [x] All plots are contour plots with outliers or pseudocolor plots.
- [x] A numerical value for number of cells or percentage (with statistics) is provided.

Methodology

Sample preparation

1. BMDCs internalization and maturation evaluation

Bone marrow-derived DCs (BMDCs) from femurs and tibias of 4-week-old C57BL/6J mice were isolated and cultured as previously reported 62. Briefly, isolated BMDCs were cultured in RPMI 1640 medium supplemented with 10% FBS, 1% penicillin/streptomycin, 20 ng/mL GM-CSF (Beyotime Biotechnology) and 10 ng/mL IL-4 (Beyotime Biotechnology). The medium was half replaced every 2 days and immature BMDCs were collected on day 6 for subsequent experiments.

To evaluate the internalization efficiency of different vaccines, 5 × 10^5 BMDCs were seeded in a 24-well plate and incubated with PBS, free-forms or nanovaccines containing Cy5-labelled gHgL, gB and gp42 (final concentrations: antigen: 4 μg/mL; CpG: 4 μg/mL; MPLA: 4 μg/mL) for 4 h, respectively. BMDCs were washed three times by PBS and the percentage of Cy5+ BMDCs was determined by CytoFLEX LX Flow Cytometer (Beckman Coulter). The data were analyzed with FlowJo software X 10.0.7 (Tree Star).

To evaluate the maturation of BMDCs induced by different vaccines, 1 × 10^4 BMDCs were seeded in a 24-well plate and incubated with PBS, free-forms or nanovaccines respectively for 24 h (final concentration: antigen: 4 μg/mL; CpG: 4 μg/mL; MPLA: 4 μg/mL). After incubation 24 h, cells were collected and blocked with anti-mouse CD16/32 for 20 min at 4°C. After three washes by PBS, cells were incubated with anti-mouse CD11c-BV650 (Biolegend), anti-mouse CD86-PE/Cy7 (Biolegend), anti-mouse CD80-PE (Biolegend) and anti-mouse MHC-II-PerCP/Cy5.5 (Biolegend) antibodies for 30 min at 4°C. Then, cells were washed with PBS three times and the population of CD80+CD86+/CD11c+ and CD86+MHC-II+/CD11c+ were determined by CytoFLEX LX Flow Cytometer (Beckman Coulter) and analyzed with FlowJo software X 10.0.7 (Tree Star).

2.Neutralization assay

All sera used in this assay were treated at 56°C for 45 min to inactivate complement, and no extra complement was added. For B cell neutralization, 20 μL of two-fold serially diluted sera (starting at 1:10) or IgG (starting at 10 mg/mL) were incubated with 20 μL CNE2-EBV-GFP at 37°C for 2 h. Then, the mixture was added to 1×10^4 EBV-negative Akata cells and incubated at 37 °C for 48 h. For epithelial cell neutralization, 50 μL two-fold serially diluted sera (starting at 1:10) or IgG (starting at 10 mg/mL) were incubated with 50 μL Akata-EBV-GFP at 37°C for 2 h. The mixture was added to 0.5 × 10^4 HNE1 epithelial cells and medium was changed after 3 h. Untreated cells served as negative controls and cells incubated with virus and sera from mice mock-immunized by PBS were used as positive controls. After 48 h incubation, infected cells were counted by a CytoFLEX LX (Beckman Coulter) and analyzed with FlowJo software X 10.0.7 (Tree Star).

3.Intracellular cytokine staining assay

C57BL/6J mice were immunized with different vaccine formulations three times at week 0, 2 and 4. At week 5, spleens from different groups were ground with a sterile syringe and the cell suspension was filtered through a 70-μm cell strainer (BD) and treated with red blood cell lysis buffer (BioLegend). After washing twice with medium, cells were seeded in round bottom 96-well plates at a density of 5 × 10^6 cells/well. After restimulation with specific antigens (2 μg gHgL, 2 μg gB or 2 μg gp42/well respectively) at 37 °C overnight, 5 μg/mL brefeldin A (BioLegend) and 2 μM monensin (BioLegend) were added to block intracellular cytokine secretion and incubated for 4 h. Then, cells were washed with PBS, blocked by Fc-blocking solution (5 μg/mL of CD16/CD32 mAb, eBioscience) and stained with antibodies including anti-mouse CD45-APC/Cy7 (BioLegend), anti-mouse CD3-FITC (BioLegend), anti-mouse CD4-AF700 (BioLegend) and anti-mouse CD8-APC (BioLegend) at 4 °C for 30 min. After washing, cells were fixed and permeabilized according to the eBioscience™ Intracellular Fixation & Permeabilization Buffer Set (Invitrogen) and stained with anti-mouse IFNγ-PE/Cy7 antibody (BioLegend), anti-mouse TNFα-BV421 antibody (BioLegend) and anti-mouse IL2-PE antibody (BioLegend) at room temperature for 30 min. Cells with no stimulation were used as a negative control, while cells stimulated with phorbol myristate acetate (PMA)-ionomycin (Sigma) were used as a positive control. The population of antigen-specific T cells was measured by a CytoFLEX LX (Beckman Coulter) and the data

was analyzed using FlowJo software X 10.0.7 (Tree Star).

4.Memory B cells and memory T cells detection
C57BL/6J mice were immunized with different vaccine formulations (NP-, Free- and Al-gHgL, NP-, Free- and Al-gB, NP-, Free- and Al-gp42, NP-, Free- and Al-cocktail) three times at week 0, 2 and 4. Splenocytes harvested at week 5 (day 35) and blood samples collected at week 12 from immunized C57BL/6J mice were treated with red blood cell lysis buffer (BioLegend) at room temperature for 10 min. Then, cells were centrifuged at 300 × g, washed twice with PBS, resuspended in PBS and stained with antibodies including anti-mouse CD45-APC/Cy7 (BioLegend), anti-mouse B220-BV605 (BioLegend), anti-mouse CD8-APC (BioLegend), anti-mouse CD4- AF700 (BioLegend), anti-mouse IgG-PerCP/Cy5.5 (BioLegend), anti-mouse CD27-BV421 (BioLegend), anti-mouse CD44-PE/Cy7 (BioLegend) and anti-mouse CD62L-PE (BioLegend)for 30 min at 4°C . The assays were performed with a CytoFLEX (Beckman Coulter), and the population of memory B cells (IgG+CD27+) and effector-memory T cells (CD44hiCD62Llow) were analyzed using FlowJo software X 10.0.7 (Tree Star).

5. Establishment of the humanized mouse model
4-week-old SCID-beige mice were irradiated (1 Gy) with a RS2000 irradiator (RAD Source) and intravenously injected with Clodronate Liposomes (YEASEN; 500 μg/mouse). 24 h later, each mouse was engrafted with 1.5×10^7 human peripheral blood mononuclear cells (PBMCs) from healthy donors. Blood samples were collected 4 weeks post engraftment and treated with red blood cell lysis buffer (BioLegend). Cells were stained with antibodies including anti-human CD45-APC/Cy7, anti-human CD19-APC, anti-human CD3-FITC and anti-mouse CD45-BV510 at 4°C for 30 min. The percentages of human CD45+ cells, B cells and T cells were determined by flow cytometry with a CytoFLEX LX instrument (Beckman Coulter) and analyzed using the FlowJo software X 10.0.7 (Tree Star).

6. Detection of human cells in peripheral blood, spleens, LNs and tumors
The spleens, LNs and tumors from different groups at week 8 or earlier (mice developed sever clinical symptoms of illness) were ground with a sterile syringe. The cell suspension was filtered through a 70-μm cell strainer (BD) and treated with red blood cell lysis buffer (BioLegend). Peripheral blood samples collected at week 8 was also treated with red blood cell lysis buffer (BioLegend). Then, the cells were centrifuged at 400 × g, washed twice with PBS, resuspended in PBS, and stained with antibodies including anti-human CD45-APC/Cy7, anti-human CD19-APC, anti-human CD3-FITC and anti-mouse CD45-BV510 at 4°C for 30 min. The percentages of human CD45+ cells, B cells and T cells were determined by flow cytometry with a CytoFLEX LX instrument (Beckman Coulter) and analyzed using the FlowJo software X 10.0.7 (Tree Star).

7.Follicular helper CD4+ T (Tfh) cell detection
C57BL/6J mice were immunized with different vaccine formulations three times at week 0, 2 and 4. At week 5, spleens from different groups were ground with a sterile syringe and the cell suspension was filtered through a 70-μm cell strainer (BD) and treated with red blood cell lysis buffer (BioLegend). Then, cells were centrifuged at 300 × g, washed twice with PBS, resuspended in PBS and stained with antibodies including anti-mouse CD45-APC/Cy7 (BioLegend), anti-mouse CD3-FITC (BioLegend), anti-mouse CD4-AF700 (BioLegend), anti-mouse CXCR5-BV421 (BioLegend) at 4°C for 30 min. After washing, cells were fixed and permeabilized according to the eBioscience™ Intracellular Fixation & Permeabilization Buffer Set (Invitrogen) and stained with anti-mouse bcl6-PerCP/Cyanine5.5 antibody (BioLegend). The assays were performed with a CytoFLEX (Beckman Coulter), and the population of Tfh cells (CXCR5+Bcl6+) was analyzed using FlowJo software X 10.0.7 (Tree Star).

8. T cell activation in humanized mice
To detect the activated T cells in spleen, the single cell suspension after treated with red blood cell lysis buffer was centrifuged at 400 × g, washed twice with PBS, resuspended in PBS, and stained with antibodies including anti-human CD45-APC/Cy7 (BioLegend), anti-mouse CD45-BV510 (BioLegend), anti-human CD3-FITC (BioLegend) anti-human CD8-PC5.5 (BioLegend), anti-human CD69-PC7 (BioLegend) and anti-human CD137-APC (BioLegend)at 4°C for 30 min. The percentages of activated (hCD69+hCD137+) hCD8+ T cells were determined by flow cytometry with a CytoFLEX LX instrument (Beckman Coulter) and analyzed using the FlowJo software X 10.0.7 (Tree Star).

9. EBV infection susceptibility of PBMCs in vitro
To analyze the EBV infection susceptibility of PBMCs in vitro, 1×10^5 PBMCs from each donor were seeded in 96-well plates. 100 μl serially diluted CNE2-EBV-GFP (started from 1×) were added into each well and incubated for 48 h. Akata B cells (EBV negative) incubated with EBV were used as the positive control. PBMCs without incubation with EBV were used as the negative control. After 48h incubation, PBMCs were washed with PBS and stained by anti-human CD19-APC (BioLegend). GFP positive CD19+ B cells represented the EBV infected B cells in PBMCs in vitro Gating strategy was shown in Figure S30A. GFP-positive infected B cells were counted by a CytoFLEX LX (Beckman Coulter) and analyzed with FlowJo software X 10.0.7 (Tree Star).

Instrument

Cells were acquired on CytoFLEX LX Flow Cytometer (Beckman Coulter).

Software

Data was analyzed using FlowJo software X 10.0.7. Graphs were compiled using GraphPad Prism 8.0

Cell population abundance

Describe the abundance of the relevant cell populations within post-sort fractions, providing details on the purity of the samples and how it was determined.

Gating strategy

1. ICS staining analysis
For the stimulated mouse splenocytes, single cells were first gated with Live-Death vs SSC-A to exclude non-viable cells. Then cells were gated by mCD45 vs SSC-A, followed by mCD45+ cells gated by mCD3 vs SSC-A. mCD4 vs mCD8 gate was next used to separate CD4+ and CD8+ T cells. IFN-γ, TNF-α and IL-2 production was then assessed in CD8+ T cells and CD4+ T cells respectively.

2. Memory cell detection

For mouse peripheral blood mononuclear cells or the splenocytes, single cells were first gated by mCD45 vs SSC-A to define lymphocytes. CD45+ cells were gated by mCD3 vs SSC-A. mCD4 vs mCD8 gate was next used to separate CD4+ and CD8+ T cells. mCD44 vs mCD62L gate were used to define memory CD4+ and CD8+ T cells respectively. For memory B cells, CD45+ cells were gated by mB220, then, B220+ cells were gated by mCD27 vs mIgG.

3. Humanized mice analysis
Single cells were first gated with hCD45 and mCD45, then hCD45+mCD45- cells were gated with hCD19and hCD3.

4. Tfh cell detection
Single cells were first gated with mCD45 vs SSC-A to difine lymphocytes. mCD45+ cells were gated by mCD3 vs SSC-A. Then mCD3+ cells were gated by mCD4. Finally, mCXCR5+mbcl6+ cells were gated from CD4+ cells.

5. T cell activation in humanized mice
Single cells were first gated with hCD45 and mCD45, then hCD45+mCD45- cells were gated with hCD3. Then hCD3+ cells were gated by hCD4+ and hCD8+. Finally, hCD69+hCD137+ cells were gated from CD8+ cells.

6.EBV infection susceptibility of PBMCs in vitro
Single cells were first gated with hCD19, then hCD19+ cells were gated with GFP.

☒ Tick this box to confirm that a figure exemplifying the gating strategy is provided in the Supplementary Information.
